# Functional lipid analysis via index-based lipidomics profile: a new computational module in LipidOne

**DOI:** 10.1093/bioinformatics/btag090

**Published:** 2026-03-01

**Authors:** Husam B R Alabed, Dorotea Frongia Mancini, Martina Pergola, Luigina Romani, Sabata Martino, Albert Koulman, Roberto Maria Pellegrino

**Affiliations:** Institute of Metabolic Science-Metabolic Research Laboratories, University of Cambridge, Cambridge, UK; Department of Chemistry, Biology and Biotechnology, University of Perugia, Perugia, Italy; Department of Chemistry, Biology and Biotechnology, University of Perugia, Perugia, Italy; San Raffaele Research Center, Sulmona, L’Aquila, Italy; Department of Chemistry, Biology and Biotechnology, University of Perugia, Perugia, Italy; Institute of Metabolic Science-Metabolic Research Laboratories, University of Cambridge, Cambridge, UK; Department of Chemistry, Biology and Biotechnology, University of Perugia, Perugia, Italy

## Abstract

**Motivation:**

Understanding the functional roles of lipids is essential for interpreting metabolic phenotypes in health, disease, and dietary interventions. However, lipidomic analyses typically focus on individual lipid species, making it difficult to extract mechanistic and systems-level insights. We therefore asked how quantitative lipidomic data can be translated into biologically structured and function-oriented interpretations.

**Results:**

Here, we present a major update to LipidOne (lipidone.eu), introducing the novel analytical module: Functional Lipid Analysis (FLA). FLA computes 42 indices describing lipid functions related to membrane structure, energy storage, and signaling. Indices are derived from lipid classes and fatty acyl-, alkyl-, and alkenyl-chain composition, statistically compared across experimental groups, and explored using multivariate and visualization tools. Each index is semantically annotated and linked to predicted protein mediators, enabling pathway- and network-based interpretation. Application to published datasets confirmed previous conclusions while uncovering additional biologically coherent functional insights.

**Availability and implementation:**

New FLA module is freely available through LipidOne.eu web platform. The LipidOne FLA core R script (v1.0.0) is archived on Zenodo (DOI: 10.5281/zenodo.18468230). The LipidOne web platform is available at https://lipidone.eu.

## 1 Introduction

Lipids constitute a highly diverse group of biomolecules that are traditionally classified into their major categories based on their structure, each containing multiple classes and subclasses of structurally related species. This complexity arises from their modular architecture: lipid molecules are assembled from distinct building blocks—such as glycerol, acyl- alkyl- alkenyl-chains, sphingoid bases, phosphate groups, and saccharides—each with a specific metabolism, enzymatic regulation, and subcellular distribution. Despite their diversity, different biosynthetic routes can converge to produce identical or functionally similar lipid molecules, reflecting a high degree of redundancy and plasticity within lipid metabolic networks (Fahy *et al.* 2005).

### 1.1 From lipid complexity to biological insight: a computational framework for functional lipidomics

The biological roles of lipids are equally diverse. They act as essential components of cellular membranes, which makes them essential to cellular structures as well as regulating membrane protein function, as reservoirs of metabolic energy, and as powerful signaling mediators involved in virtually every physiological process. Importantly, lipid functions can be exerted at multiple levels: entire classes (e.g. triglycerides, phospholipids), individual molecular species (e.g. PE 16:0/22:6), or even specific substructures such as fatty acyl chains or headgroups may carry distinct and sometimes independent biochemical implications (Conroy *et al.* 2024).

Lipids are highly dynamic molecules that undergo continuous remodeling in response to environmental, physiological and pathological stimuli. Compared with aqueous (polar) metabolites, which can fluctuate rapidly and transiently, lipid species generally change on slower timescales when considering membrane composition and neutral-lipid storage/usage. Nevertheless, specific lipid signaling steps can be fast (e.g. COX- and LOX-mediated eicosanoid production) and also the localization of lipid in membranes can be highly dynamic. Because lipids frequently sit at entry and exit nodes of complex metabolic networks, they integrate upstream and downstream biochemical signals; the cumulative effects of these changes are particularly relevant to chronic diseases and long-term adaptation to physiological stress (Cho *et al.* 2023).

Moreover, lipid biochemistry is tightly interwoven with protein signaling pathways and the metabolism of water-soluble metabolites, forming a complex regulatory network that governs cell fate decisions, membrane organization, and energy homeostasis (Sych *et al.* 2022).

Over the past decade, lipidomics has emerged as a powerful omics approach to quantitatively map the lipid composition of biological systems. Typically, a lipidomic study focuses on one or more of the following applications:

- Biomarker discovery using statistical and bioinformatic analysis, to identify lipid-based markers for early diagnosis, disease progression, or therapeutic response—especially valuable in cardiometabolic and neurodegenerative disorders (Hornemann 2022).
- Pathway analysis, which seeks to reconstruct lipid transformation routes and infer the involvement of specific genes, enzymes, and regulatory proteins by integrating lipidomic profiles with transcriptomic or proteomic data (Nguyen *et al.* 2017).
- Functional analysis, which focuses on interpreting the biological roles of lipids based on their variation across conditions, and on linking those changes to cellular behaviors such as membrane remodeling, energy shifts, or inflammatory signaling.

However, unlike biomarker discovery and pathway reconstruction, functional interpretation remains the main bottleneck in the utilization of lipidomics. The enormous diversity of lipid species and the limited coverage of curated knowledge bases (KEGG, Reactome, WikiPathways, HMDB) leave many measured species unmapped, hampering downstream functional inference. Existing platforms such as LipidOne (Pellegrino *et al.* 2022, Alabed *et al.* 2024) and BioPAN (Gaud *et al.* 2021) mitigate pathway gaps.

When it comes to functional analysis, it is essential to recognize that lipids perform three fundamental biochemical functions in living organisms: structural, energetic, and signaling/regulation (Uzman 2001). This triad is a foundational concept in lipid biochemistry, but it has not yet been fully translated into computational tools capable of resolving which of these functions are altered in a given experimental context (Shevchenko and Simons 2010). A few platforms, such as LION/web (Molenaar *et al.* 2019), MetaboAnalyst (Lu *et al.* 2023) and LipidSig (Lin *et al.* 2021), are excellent resources that offer, among other features, tools described as “functional analysis” for lipidomic datasets. However, in these contexts, “functional analysis” is most often implemented through category/ontology- or property-based annotations and enrichment-style summaries, which are highly useful but typically yield set-level outputs rather than a compact panel of directional, quantitative functional readouts.

This gap is especially relevant because lipid metabolites, due to their chemical heterogeneity, functional redundancy, and compartmental specificity, require dedicated analysis strategies. Interpreting lipidomic changes purely in terms of class enrichment or fold changes is often insufficient and may lead to misleading conclusions. This is particularly true for functional analyses that adopt enrichment-based strategies originally developed for gene expression data. As highlighted by Lee *et al.* such approaches can misrepresent the biology of small molecules like lipids, which differ substantially from genes in structure, dynamics, and regulation (Lee *et al.* 2025). In other words, most existing platforms excel at annotating and enriching lipid lists, but they do not provide a compact, quantitative representation of lipid functions that can be directly used as input for downstream statistical modelling.

What is needed is a framework that integrates quantitative lipid features, biochemical functions, and systems-level reasoning to generate interpretable biological outputs in the form of functional readouts, rather than category-level summaries.

To address this unmet need, we developed a dedicated module within the LipidOne platform that systematically translates quantitative lipidomic data into biochemically interpretable functional profiles. The new functional lipid analysis (FLA) module provides a structured and reproducible approach to identify whether specific lipid functions are enhanced or suppressed across experimental conditions by summarising each lipidomic profile into a small set of biochemically interpretable functional variables that can then be analysed with standard statistical tools. These statistical tools (differential analysis, PCA/PLS-DA, clustering) are similar to those implemented in other lipidomics platforms; the distinctive feature of FLA is the functional layer on which they operate.

## 2 Methods

### 2.1 Functional lipid analysis (FLA) module

The FLA module within LipidOne is based on a curated set of 42 mathematically defined indices, which are quantified from user-uploaded lipidomics datasets. These indices are calculated as ratios between lipid species, subclasses, or defined building blocks—including acyl, alkyl, or alkenyl chains, and headgroups. Each index is specifically designed to capture distinct biochemical alterations in lipid function, reflecting properties such as the saturated/unsaturated lipid ratio, triglyceride metabolism, or the relative abundance of signaling lipids like ceramides.

### 2.2 Construction and curation of the functional index library

The FLA module is built on a library of 42 functional lipid indices defined as ratios or weighted sums of lipid classes, molecular species or fatty-acyl building blocks (Table 1). The initial set of candidates was assembled by a targeted survey of the lipidomics and lipid biochemistry literature (PubMed) using combinations of terms such as “lipid index,” “lipid ratio,” “membrane fluidity,” “PUFA/SFA,” “n-6/n-3,” “lysophospholipid signalling” and “ceramide.” Indices were retained when they had a clear biochemical or biophysical rationale (e.g. membrane fluidity, mitochondrial inner-membrane composition, neutral lipid storage versus mobilization), could be unambiguously expressed in terms of standard lipid classes/species or chain descriptors, and were linked by at least one primary study or review to a biological function, phenotype or pathway. In other cases, the literature converged on qualitative contrasts such as “storage versus membrane lipids” or “ω-6 versus ω-3 tone” without specifying a unique formula; here we designed new composite indices that formalise these contrasts into explicit ratios, guided by expert knowledge in lipid biochemistry. All 42 indices were thus defined a priori on biochemical grounds, without data-driven feature selection or optimisation on the case studies analysed in this work.

**Table 1:** Functional lipid indices, biochemical classification, and calculation formula.

Functional Category	Index Name	Formula	Proteins	Reference
Structural	Cardiolipin Fraction (Note 1)	(CL/(Total − CL))	TAZ, CRLS1, ACAD9	(Kagan *et al.* 2016)
	CE/Total Index	(CE/(Total − CE))	ACAT1, SOAT1, CETP	(Barter *et al.* 2003, Chang *et al.* 2009, Bernstein *et al.* 2013)
	Chol/PLmem Index	(Cholesterol/(PC + PE + PS + PI + SM))	ABCA1, HMGCR, LCAT, LDLR, NPC1, NPC2, SGMS1, SGMS2, SMPD1, SOAT1	(Van Meer *et al.* 2008, Subczynski *et al.* 2017)
	Chol/SM Index	(Cholesterol/SM)	ABCA1, HMGCR, SGMS1, SGMS2, SMPD1	(Ramstedt and Slotte 1999, Slotte 1999)
	Double Bond Index (Note 2)	(Σ intensity × DB)/(Σ intensity)	FADS1, FADS2, SCD1, ELOVL5	(Stillwell and Wassall 2003, Weiss-Hersh *et al.* 2020, Baccouch *et al.* 2023)
	Mono/Poly Ratio	(MUFA/PUFA)	FADS1, FADS2, SCD1, SCD2, ELOVL5	(Ntambi 1999, Baccouch *et al.* 2023)
	Odd/Even Chain	(Odd‑chain/Even‑chain)	HACL1, ECHDC1, ACAT2, ACAD8, MBOAT1, DHDDS	(Escribá *et al.* 2015, Benzerouk *et al.* 2020)
	OxPL/PL Index (Note 3)	(OxPL/(PL − OxPL))	ALOX15, LTA4H, PLA2G4A, GPX4, PTGS1	(Fruhwirth *et al.* 2007, Itri *et al.* 2014)
	PC Unsat/Sat Index	(PC unsaturated/PC saturated)	FADS1, FADS2, SCD1, ELOVL5, PPARA	(Stillwell and Wassall 2003, Róg *et al.* 2004)
	PE/PC Index	(PE/PC)	LPCAT3, GPAM, PLD1, GPCPD1, GPX4	(Li *et al.* 2006, Gimeno and Cao 2008)
	Membrane Fluidity Index	PC/(PE + SM)	PCYT1A, CEPT1, PCYT2, SGMS1, SMPD1, PEMT	(Fajardo *et al.* 2011)
	PL/SM Index	(PL/SM)	SMPD1, SMS1, SMS2, SGMS1	(Simons and Ikonen 1997, Lingwood and Simons 2010, Fajardo *et al.* 2011)
	Saturation Index	(Saturated/Unsaturated)	SCD1, FADS1, FADS2, ELOVL5, SCD2	(Stillwell and Wassall 2003, Weiss-Hersh *et al.* 2020)
	SM/PC Index	(SM/PC)	SMase, SCD1, FASN, SGMS1, CDIPT	(Simons and Ikonen 1997, Lingwood and Simons 2010, Fajardo *et al.* 2011)
	Structural/Energetic (Note 4)	((PC + PE + SM)/(TG + DG))	CPT1A, SCD, ACADM, PDK1, FASN	(Fahy *et al.* 2009)
Signaling	(LPC+LPE)/PL Index	((LPC + LPE)/PL)	PLA2G4A, LPCAT3, PLD1	(Lin and Boyce 2006, Meyer Zu Heringdorf and Jakobs 2007, Shao *et al.* 2018, Yaginuma *et al.* 2023, Henze *et al.* 2025)
	AA/DHA Index	(AA/DHA)	PLA2G4A, ALOX5, PTGS2, PTGDS, CYP1B1	(Serhan *et al.* 2008, Calder 2013)
	BMP Fraction (Note 5)	(BMP/(Total − BMP))	NPC1, SMPD1, SCARB2	(Hullin-Matsuda *et al.* 2009)
	Cer/SM Index	(Ceramide/Sphingomyelin)	CerS1, CerS2, SMS1, SMS2, SPTLC1	(Hannun and Obeid 2018, Ogretmen 2018, Stith *et al.* 2019, Thakkar *et al.* 2025)
	Ceramide Fraction (Note 6)	(Ceramide/(Total − Ceramide))	CerS1, SMS1, CerS2, SGMS2, LASS1	(Stith *et al.* 2019, Thakkar *et al.* 2025)
	DG/PL Index	(DG/PL)	PLCB1, PLCD1, PLCG1, PRKCA, PRKCB	(Berridge 1987, Newton 2010)
	Ether Lipid Fraction (Note 7)	(Ether lipids/(Total − Ether))	ELOVL1, PPARA, PLA2G4A, GPD1L, LPCAT3	(Dean and Lodhi 2018, Fontaine *et al.* 2020, Rangholia *et al.* 2021)
	Ferroptosis Susceptibility Index (Note 8)	(PL containing 20:4, 22:4 or 22:6)/(Total − same PL)	GPX4, ACSL4, FTH1, TFRC, SLC7A11, ALOX12, NCOA4, FSP1	(Doll *et al.* 2017, Lee *et al.* 2021, Liu *et al.* 2024)
	GM3/GM2 Ratio (Note 9)	(GM3/GM2)	ST3GAL5, HEXA, HEXB	(Sachinidis *et al.* 1996, Tajima *et al.* 2023)
	LPC/PC Index	(LPC/PC)	PLA2, PLD1, LCAT, GPAM, GPCPD1	(Prokazova *et al.* 1998, Law *et al.* 2019)
	LPE/PE Index	(LPE/PE)	LPCAT3, GPCPD1, PLD2, GPD1, LPAAT	(Hisano *et al.* 2021, Yamamoto *et al.* 2022)
	Lyso-O/PL-O (matched)	((LPC‑O + LPE‑O)/(PC‑O + PE‑O)) with chain‑matching	AGPS, GNPAT, LPCAT2, LPCAT3, PLA2G6, PLA2G7	(Yang *et al.* 1996, Werner *et al.* 2020)
	Lyso-P/PL-P (matched)	((LPC‑P + LPE‑P)/(PC‑P + PE‑P)) with chain‑matching	AGPS, FAR1, GNPAT, PLA2G6, TMEM189	(Farooqui 2010, Werner *et al.* 2020)
	Lyso/PL (matched)	((LPC + LPE)/(PC + PE)) with chain‑matching	PLA2, LPL, ABCA1, LPL, ALOX5	(Yang *et al.* 1996, Farooqui 2010, Dennis *et al.* 2011, Murakami *et al.* 2020, Werner *et al.* 2020)
	PA/PL Index	(PA/PL)	DGKZ, LPIN1, MTOR, PLD1, PLD2	(Hornberger *et al.* 2006, Foster *et al.* 2014)
	PI/PL Index	((PI + PI‑O)/(PC + PE + PG + PS))	PIK3CA, PIK3CB, PLCG1, PLCG2, INPP5D, PTEN	(Di Paolo and De Camilli 2006)
	ω6/ω3 Index	((18:2 + 20:4)/(18:3 + 22:6))	FADS1, FADS2, ELOVL5, ELOVL2, ACAT2, ALOX5	(Simopoulos 2008, Calder 2017)
Energy	Acylcarnitine Fraction (Note 10)	(CAR/(Total − CAR))	CPT1A, CPT2, SLC25A20	(Bartlett and Eaton 2004)
	DG/TG Index	(DG/TG)	ACSL1, DGAT1 DGAT2, LPL, AGPAT2, LCAT	(Reue and Brindley 2008, Lass *et al.* 2011)
	Energy Load Index (Note 11)	((TG + DG + CE)/(Total − TG − DG − CE))	DGAT1, DGAT2, ACSL4, CPT1A	(Listenberger *et al.* 2003)
	Long/Medium Chain	(Long chains/Medium chains)	CPT1A, ACADM, ACADL, ACAT2, PDK4	(Papamandjaris *et al.* 1998, Nakamura *et al.* 2014, Mett *et al.* 2021)
	Neutral/Polar (Note 12)	(Neutral lipids/Polar lipids)	ABCA1, NPC1, FABP4, NPC2, AP2M1	(Harayama and Riezman 2018)
	Short/Long Chain	(Short chains/Long chains)	ACSL1, ACADM, ECHDC1, MBOAT1, LPL	(Schönfeld and Wojtczak 2016)
	Storage Index (Note 13)	(TG/(Total − TG))	DGAT1, ACAT1, FASN, ACSL1, LPL	(Samra 2000, Walther and Farese 2012, Czech *et al.* 2013)
	TG/CE Index	(TG/CE)	ACSL1, ACSL4, DGAT1, DGAT2, LPL	(Maxfield and Tabas 2005, Farese and Walther 2009, Walther and Farese 2012)
	TG/FA Index	(TG/FA)	ACSL1, DGAT1, DGAT2, LIPE, PNPLA2	(Zimmermann *et al.* 2004, Yen *et al.* 2008)
	TG/PL Index	(TG/PL)	DGAT1, DGAT2, LPL	(Zimmermann *et al.* 2004, Moessinger *et al.* 2014)

Notes:

(1) Cardiolipin Fraction: cardiolipins (CL) over total lipid content minus CL.

(2) Double Bond Index: weighted average number of double bonds per molecule, calculated as the sum of (abundance × DB count) divided by total abundance.

(3) OxPL/PL Index: oxidized phospholipids (OxPL, or species with ‘; O’ suffix) over total non-ether phospholipids (PC, PE, PG, PI, PS).

(4) Structural/Energetic: ratio between structural lipids (PC, PE, SM) and energetic lipids (TG, DG).

(5) BMP Fraction: bis(monoacylglycero)phosphate (BMP) over total lipid content minus BMP.

(6) Ceramide Fraction: ceramide abundance over total lipid content minus ceramides.

(7) Ether Lipid Fraction: sum of ether lipids (PE O, PC O, LPC O, LPE O) divided by the total lipid content minus ethers.

(8) Ferroptosis Susceptibility Index: includes phosphatidylethanolamines (PE) and plasmalogen forms (PE P) containing polyunsaturated fatty acids (20:4, 22:4, or 22:6). These species are prone to lipid peroxidation and are key drivers of ferroptosis.

(9) GM3/GM2 Ratio: ratio between gangliosides GM3 and GM2, when both are detected.

(10) Acylcarnitine Fraction: acylcarnitines (CAR) over total lipid content minus CAR.

(11) Energy Load Index: sum of TG, DG, and CE over total lipids excluding these three.

(12) Neutral/Polar: ratio between neutral lipids (TG, CE) and polar lipids (PC, PE, PG, PI, PS, SM, LPC, LPE, PE O).

(13) Storage Index: triacylglycerol (TG) abundance over total lipid content minus TG.

Each index is specified at a defined structural level, and lipid membership in its numerator and denominator is determined by rule-based mapping of user-provided identifiers to an internal LIPID MAPS-compatible ontology. Class labels (e.g. PC versus LPC, TG versus DG), acyl/alkyl/alkenyl chains for chain-level indices, and the annotation level chosen by the user (molecular species versus sum composition) jointly determine whether a lipid contributes to a given index. When only sum compositions are available, indices requiring full chain resolution are automatically disabled, while class-level indices remain computable. These rules are encoded in the LipidOne back-end and applied systematically to all datasets.

For each index, we also compiled a set of proteins that directly catalyse, regulate or consistently respond to the lipid species involved, including acyltransferases, lipases, desaturases, elongases, transporters and key regulatory enzymes. Protein lists were curated by combining pathway resources (e.g. KEGG, Reactome, LIPID MAPS pathway annotations) with the primary literature cited in Table 1, and proteins were included only when a direct mechanistic link to the index lipids could be established.

Index–protein associations were curated in human and then extended to the ten model organisms supported by LipidOne using STRING orthology mapping, propagating links only when at least one clear ortholog was available (summarised in Table S2). Because lipid metabolism diverges across kingdoms, especially between animals, plants and fungi, we recommend interpreting protein-level suggestions more cautiously in non-mammalian systems and primarily as hypotheses for downstream validation.

LipidOne input conventions, including supported shorthand nomenclature, lipid class coverage, and annotation levels, have been described previously (Alabed *et al.* 2024) and are referenced from the LipidOne homepage. All datasets must be successfully uploaded to LipidOne and validated using these standard conventions prior to FLA. For datasets generated using vendor-specific nomenclature that differs from LIPID MAPS shorthand, LipidOne provides a Shorthand Lipid Translator utility to convert common identifiers into the required format. For FLA, only lipid classes and acyl chain compositions listed in Table 1 contribute to each functional index; indices are not computed when the required lipid species are absent.

To facilitate interpretation, the indices are grouped into three major functional categories, corresponding to the principal roles of lipids in biological systems (Gurr *et al.* 2016):

Structural indices, which describe both quantitative and qualitative features of membrane-associated lipids. These indices capture variations in the abundance of entire lipid classes (e.g. phosphatidylcholines, cardiolipins), but also reflect molecular-level properties such as the degree of unsaturation (linked to membrane fluidity), average chain length (affecting membrane curvature), and the presence of ether bonds. While certain structural lipids like cardiolipins are localized in mitochondria and have multifunctional roles, the structural indices in this group aim to represent the overarching biophysical properties of lipid assemblies across different cellular compartments.

Signaling indices, which track lipid species and subclasses involved in cell signaling, inflammation, and intercellular communication. These include not only individual bioactive molecules—such as omega-3 and omega-6 polyunsaturated fatty acids and eicosanoid precursors—but also broader lipid classes or sub-classes known for their signaling functions, including ceramides, lysolipids, and ether lipids. These molecules act as metabolic messengers, modulating immune responses, stress signaling, and pathways related to cell growth, differentiation, or apoptosis.

Energy-related indices, which capture lipid species involved in metabolic energy storage, mobilization, and mitochondrial function. These include not only neutral lipids such as triglycerides and diglycerides, but also lipid classes and acyl chain features linked to β-oxidation. For instance, changes in acyl chain length may reflect enhanced or impaired fatty acid catabolism, while specific mitochondrial lipids—such as cardiolipins and acyl-carnitines—are functionally associated with energy production and transport (Grevengoed *et al.* 2014, Paradies *et al.* 2019). Although some of these, like cardiolipins, may also contribute to structural integrity, their metabolic implications justify their partial inclusion in this group.

Table 1 lists all indices along with their associated functional category and the formula used to calculate their numerical values.

Table S1 provides the full specification of the FLA index library, including each index formula, the associated enzyme list, the biochemical description, and the standardized interpretative phrases used by LipidOne for EXP ≪ CTRL and EXP ≫ CTRL.

Each index in Table 1 has been systematically curated based on evidence from the scientific literature and reflects established relationships between lipid composition and cellular functions. Indices are also linked to specific enzymes or regulatory proteins that determine, modulate or respond to lipid variation. For instance, the Ceramide/Sphingomyelin Index is associated with enzymes involved in apoptotic signaling and stress responses such CerS1, CerS2, SMS1, SMS2, SPTLC1, while the DG/TG Index reflects lipid mobilization and energy storage dynamics by activation of ACSL1, DGAT2, LPL, AGPAT2, LCAT proteins. By linking these indices with the associated proteins, LipidOne the opportunity to make inferences about the mechanisms that drive the differences between the analyzed samples.

As the indices are directly related to specific proteins the FLA module facilitates the construction of protein interaction networks, based on predicted activations or deactivations of these proteins, allowing researchers to visualize how specific lipid alterations might influence broader biological pathways. The network-based interpretation extends to protein clusters, where specific protein families—such as kinases or phosphatases—may be identified as central mediators of lipid-driven pathways. By visualizing these interactions, users can generate hypotheses regarding the functional consequences of lipidomic changes. These insights can then inform experimental designs or suggest potential therapeutic interventions.

Finally, since LipidOne has been designed to build protein interaction networks for 10 model organisms, we provide homologous proteins from these species based on their short names in the STRING database. Table S2 reports the curated index–protein mapping in human and its orthology-based propagation across the supported model organisms (STRING ortholog mapping), indicating which indices retain protein support in each species.

The full set of 42 curated functional indices is automatically computed for each sample in the lipidomics dataset when the user accesses the FLA module of LipidOne. FLA operates on the lipid names contained in the user input data matrix and extracts class and chain information using a rule-based parser. Class-level indices (e.g. LPC/PC, TG/CE, Structural/Energetic) are computed whenever the relevant lipid classes are present, irrespective of whether lipids are reported as molecular species or sum compositions. Indices that require explicit fatty-acyl chains (e.g. AA/DHA, n-6/n-3, specific ferroptosis-related chains) are computed only for samples in which the corresponding chains can be identified in the lipid names; when the required components are absent or their denominator is zero, the index value is set to missing (NA) and will not be displayed. Thus, datasets reported purely at sum-composition level yield a reduced subset of FLA indices, whereas molecular-species and mixed datasets allow a larger fraction of indices to be calculated, without inferring any chain composition beyond what is provided.

The use of ratio-based definitions ensures that the indices remain interpretable, scalable, and robust across experimental platforms, minimizing dependency on absolute quantification. The resulting numerical values serve as variables for statistical analysis and group comparison enabling the identification of biologically meaningful differences across experimental conditions, as illustrated in the following sections.

### 2.3 From functional indices to predicted protein networks

In FLA, the link between functional lipid indices and enzyme-level hypotheses is explicitly reaction-based and follows the same philosophy as the predicted protein network that we previously described for pathway-based analyses of lipid classes, molecular species and lipid building blocks in LipidOne (Alabed *et al.* 2024). In that setting, significantly altered lipids are mapped to the enzymes that catalyse reactions among lipids, and these enzymes are then used as seeds for a STRING interaction network. Here we generalise this framework by using functional indices as the starting variables. For each enzyme, we consider the set of lipid reactions it catalyses and, for each group, compute aggregate “substrate” and “product” weights by summing the abundances of the corresponding lipids. For a simple reaction A + B → C + D, this corresponds to comparing, between experimental and control groups, the combined abundance of A and B with that of C and D. When the product-to-substrate balance shifts toward products in the experimental group (relative to control), we interpret this pattern as a net forward drive through the reaction and assign the enzyme an activity-oriented increase; the opposite pattern suggests reduced net flux. Importantly, FLA does not estimate protein concentrations and does not provide a direct kinetic measurement of enzyme activity. Instead, it uses curated stoichiometric relationships between lipids and the observed lipid phenotype to map, for each enzyme, an activity-oriented direction of change that is mechanistically consistent with the underlying reaction network. The same reaction-based mapping has been implemented in LipidOne for class-, species- and building block-based analyses since its initial release; the FLA module reuses this framework but replaces individual lipids with functional indices as the input layer, after assigning each index to its curated set of enzymes as described above.

### 2.4 FLA workflow

The FLA module integrates statistical and visualization tools to detect and interpret alterations in lipid function across experimental conditions. All analyses are implemented in R and executed server-side, ensuring reproducibility, transparency, and scalability. Starting from the user’s lipidomics dataset, the platform computes a panel of 42 mathematically defined functional indices, which act as the primary variables. These indices are statistically compared across groups (typically using *t*-tests or ANOVA, depending on the number of groups), and the resulting log_2_ fold changes and *P*-values drive the plots and summaries. Unless otherwise stated, *P*-values reported for FLA indices are unadjusted; given the limited number of pre-defined indices tested (≤42), we report raw *P*-values for interpretability. An application of these analyses is shown further below in the text.

As the general philosophy of LipidOne, the tools of FLA module, have been grouped into four categories:

#### 2.4.1 Univariate statistical analysis

**Figure 1:**
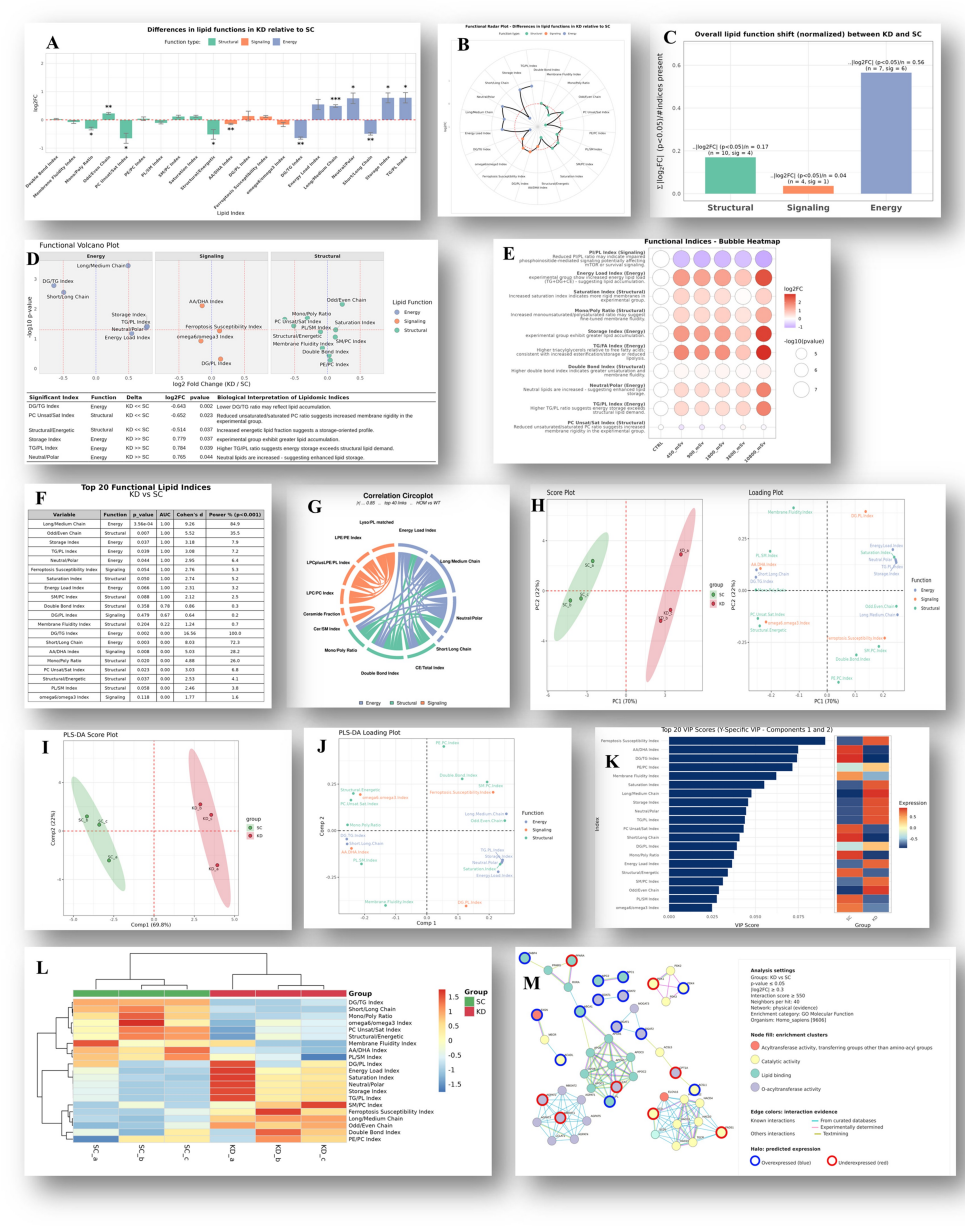
Examples of graphical outputs generated by the FLA module in LipidOne. (A) Bar plot comparing all indices; (B) Radar plot summarizing the overall functional landscape; (C) Functional Dominance plot showing a three-bar panel (Structural, Signaling, Energy) to study functional shifts within a study; (D) Functional Volcano plot; (E) Functional Bubble plot displaying a bubble heatmap of top index differences across groups; (F) Functional biomarker discovery table; (G) Functional Correlation Circos plot; (H) PCA score and loading plots; (I) PLS-DA score plot; (J) PLS-DA loading plot; (K) VIP score bar plots; (L) Functional heatmap of indices; (M) Predicted functional protein network.

**Functional summary:** Compares all indices between two selected groups. Bar Plot show the group mean log2 fold change, while the error bars depict the standard error of the log2 fold change estimated by first-order error propagation (delta method) from within-group variability and sample size. This Standard Error-based approach is coherent with the Welch test used for significance and provides a compact, distribution-light estimate of uncertainty. Bars are colored according to the predominant lipid function of each index (Fig. 1A). Radar Plot show a companion radar chart summarizes the overall functional landscape (Fig. 1B). Functional Dominance show a three-bar panel (Structural, Signaling, Energy) that reports, at a glance, the prevailing functional shift (Fig. 1C). For each function, we aggregate the magnitude of change by summing the absolute log2FC of indices that pass significance and normalizing by the number of indices effectively evaluated in that function, to avoid bias from missing/filtered indices. Larger bars indicate a stronger net deviation of that function in the experimental group. Functional Summary outputs are accompanied by CSV exports reporting per-index statistics (log2FC, SE, *P*-value from Welch’s test, and other metrics when available). For indices that meet significance thresholds, the CSVs also include a standardized Interpretation field with concise phrases tailored to the index identity and direction of change, enabling immediate reuse in figure tables and narrative summaries.

**Functional volcano plot**: Highlights the magnitude of change (log_2_ fold change) and significance for each index. In FLA, a three-panel volcano separates indices by functional category. A results table lists indices that are both significant and beyond the user-defined variation threshold (Fig. 1D). The significant indices are presented together with brief sentences explaining the biochemical meaning of the differences.

**Functional bubble plot:** Displays a bubble heatmap of the top ten indices across groups; bubble size reflects significance and color encodes fold change. Each index is accompanied by a concise interpretive sentence (Fig. 1E).

**Functional biomarker discovery**: Ranks indices by discriminative performance (*P*-value, ROC AUC—polarity-corrected when needed—statistical power, Cohen’s d). Outputs a Top-20 table (CSV + PNG) for rapid screening and prioritization (Fig. 1F).

**Functional correlation circoplot**: Visualizes pairwise correlations between indices to reveal co-regulation and functional synergy (Fig. 1G).

#### 2.4.2 Multivariate statistical analysis

Instead of analyzing hundreds or thousands of molecular lipid species, FLA works with a compact set of functional indices (currently 42) that explicitly encode lipid roles (structural, energetic, signaling). This biochemistry-driven reduction in data complexity makes principal components and latent variables easier to interpret, because each feature reflects a function rather than an individual species and reduces co-linearity that often occurs in lipidomics data. By aggregating related species, the indices improve signal-to-noise and often sharpen group separation in PCA and PLS-DA. (see details in in the results section). Finally, VIP scores and loadings calculated on indices map directly onto functional biochemical mechanisms, revealing unexpected links between lipid functions.

**Functional PCA:** PCA on the matrix of functional indices (centered and auto-scaled by default; scaling options are user-configurable). Produces score plots (sample clustering) and loading plots (index contributions), enabling dimensionality reduction and identification of dominant functional axes (Fig. 1H).

**Functional PLS-DA:** Performs Partial Least Squares Discriminant Analysis on the functional index matrix. Outputs include score plots with confidence ellipses (Fig. 1I), loading plot (Fig. 1J), and VIP score bar plots (Fig. 1K). This supervised method improves discrimination between experimental groups and highlights key functional biomarkers.

#### 2.4.3 Clustering analysis

**Functional heatmap:** Creates a clustered heatmap of indices across all samples of groups selected (Fig. 1L). Hierarchical clustering is applied to both samples and indices. User can select the number of indices to include and apply one of seven diverse algorithms of distances, aiding in the identification of group-specific functional or nutritional profiles and outlier patterns.

#### 2.4.4 Lipid system biology

**Predicted functional proteins network:** This tool links each lipid functional index to relevant proteins and enzymes using the STRING database (Fig. 1M). The resulting network graph highlights biological pathways and regulatory modules, with nodes colored according to cluster annotations, thus providing a mechanistic link between lipidomic alterations and the proteins potentially involved in their regulation or response. To enhance interpretability and flexibility, the user can adjust key parameters of the analysis, including the statistical thresholds used to define significant indices (*P*-value and log_2_ fold change), the type of STRING interaction network to be queried (full network or physical interactions only), and the confidence score threshold for STRING interactions. Additionally, users can specify the number of neighboring proteins to retrieve for each lipid-associated hit and choose the annotation system used to color the protein nodes in the network. Available annotation sources include Gene Ontology (Biological Process, Molecular Function, and Cellular Component), KEGG Pathways, Pfam Domains, InterPro Families, UniProt Keywords, and Reactome Pathways. This high degree of configurability allows users to tailor the network reconstruction to the specific biological context of their study, supporting hypothesis generation and functional interpretation of lipidomic data.

## 3 Results

To demonstrate the ability of the FLA module to detect biologically meaningful alterations in lipid metabolism, we applied it in two independent lipidomic datasets: one investigating the role of PDK1 in cardiomyocyte lipid utilization (Atser *et al.* 2025) and another characterizing lipidomic signatures associated with hepatocellular carcinoma and chronic hepatitis C virus–related conditions (Caponigro *et al.* 2023). Detailed analysis reports are provided in the Supplementary Materials (see the Case studies using Functional Lipid Analysis section).

FLA turns untargeted lipidomes into mechanistically interpretable readouts with organellar context. Rather than listing hundreds of species, FLA quantifies a curated set of functional indices spanning signaling, structural, and energy domains and ties each index to a concise biochemical interpretation. The result is an analysis that tells not only what changed, but what it may imply functionally. Where enzyme or organelle processes are mentioned, these should be interpreted as hypotheses inferred from index behavior and STRING-contextualization, not direct measurements. FLA is not an enrichment analysis; it is a hypothesis-generating, mechanistically informed interpretation based on quantitative lipid features and prior biochemical knowledge.

Across two independent case studies, FLA both replicated the key findings of the original works and extended them with testable hypotheses that are difficult to obtain from class-level summaries alone. In cardiomyocytes, FLA separated storage-positive from mobilization-negative indices and mapped the phenotype onto a Pdk1–Pdk4–Pdha1 network interfacing with Pnpla2 (ATGL) and FAO enzymes. In HCV/HCC plasma, FLA confirmed lysophospholipid depletion in HCC and newly highlighted ether-lysophospholipid turnover, putative mitochondrial/inner-membrane remodeling signals (LPE/PE), and ceramide-axis differences. Because indices are ratio-based and statistics are attached to each readout (effect size, AUC, power), the outputs are interpretable, portable, and biomarker-ready.

Importantly, FLA links statistical outputs to standardized biochemical and mechanistically oriented statements, converting lipidomic ratios into actionable interpretations that are accessible to clinical and translational teams without deep lipid biochemistry expertise.

To our knowledge, FLA is the first general-purpose bioinformatics module that goes beyond lipid ontology/characteristic enrichment by (i) organizing lipidomes into directional functional axes supported by quantitative indices, (ii) overlaying significant functional signals onto protein interaction networks to propose enzyme-level hypotheses, and (iii) delivering a compact suite of publication-grade visuals (summary bars, bubble heatmap, functional volcano, top-index table, protein network) in minutes. This positions lipidomics not only as a descriptive assay but as a strategic tool for mechanism discovery and translational biomarker development.

## 4 Limitations and future directions

Accurate computation of several indices requires molecular-species–level annotation (chain length/unsaturation and, where relevant, O-/P-linkages). Datasets reported only as sum compositions limit index coverage.

Index accuracy depends on class coverage and identification quality. Ratio-based design mitigates but does not eliminate variability due to extraction, chromatography, adduct handling, or missing values (zero handling can inflate ratios at low abundance).

Some indices are correlated by construction; they should be interpreted as a panel rather than independent tests. Statistical control remains essential.

FLA infers function from correlations in lipidomes and maps results onto STRING; it does not measure enzyme activity. Network-level hypotheses (e.g. ATGL, SCD1, ELOVLs) require orthogonal validation (proteomics, flux assays).

Finally, the index library is curated and finite: rare contexts or novel lipid chemistries (e.g. oxidized species, cardiolipin microheterogeneity) may not yet be captured.

Future work will expand the index library (including oxidized lipids and pathway-specific composites), add data-driven index discovery and uncertainty quantification, tighten cross-study harmonization (QC rules, FDR defaults, zero-handling), and deepen multi-omics integration (proteome, transcriptome, 13C-flux) so that FLA can progress from functional readouts to testable, enzyme-level mechanisms at scale.

## Supplementary Material

btag090_Supplementary_Data

## Data Availability

The data used in this article were derived from previously published studies, which are cited within the manuscript. The original data are available from the respective publications and associated repositories as described therein.

## References

[btag090-B1] Alabed HBR , Mancini DF, Buratta S et al LipidOne 2.0: a web tool for discovering biological meanings hidden in lipidomic data. Curr Protoc 2024;4:e70009. 39301800 10.1002/cpz1.70009

[btag090-B2] Atser MG , Wenyonu CD, Rowe EM et al Pyruvate dehydrogenase kinase 1 controls triacylglycerol hydrolysis in cardiomyocytes. J Biol Chem 2025;301:108398. 40074083 10.1016/j.jbc.2025.108398 PMC11999607

[btag090-B3] Baccouch R , Shi Y, Vernay E et al The impact of lipid polyunsaturation on the physical and mechanical properties of lipid membranes. Biochim Biophys Acta Biomembr 2023;1865:184084. 36368636 10.1016/j.bbamem.2022.184084

[btag090-B4] Barter PJ , Brewer HB, Chapman MJ et al Cholesteryl ester transfer protein: a novel target for raising HDL and inhibiting atherosclerosis. Arterioscler Thromb Vasc Biol 2003;23:160–7. 12588754 10.1161/01.atv.0000054658.91146.64

[btag090-B5] Bartlett K , Eaton S. Mitochondrial β‐oxidation. Eur J Biochem 2004;271:462–9. 14728673 10.1046/j.1432-1033.2003.03947.x

[btag090-B6] Benzerouk F , Djerada Z, Bertin E et al Contributions of emotional overload, emotion dysregulation, and impulsivity to eating patterns in obese patients with binge eating disorder and seeking bariatric surgery. Nutrients 2020;12:3099. 33053641 10.3390/nu12103099 PMC7650699

[btag090-B7] Bernstein DL , Hülkova H, Bialer MG et al Cholesteryl ester storage disease: review of the findings in 135 reported patients with an underdiagnosed disease. J Hepatol 2013;58:1230–43. 23485521 10.1016/j.jhep.2013.02.014

[btag090-B8] Berridge MJ. Inositol trisphosphate and diacylglycerol: two interacting second messengers. Annu Rev Biochem 1987;56:159–93. 3304132 10.1146/annurev.bi.56.070187.001111

[btag090-B9] Calder PC. Omega-3 fatty acids and inflammatory processes: from molecules to man. Biochem Soc Trans 2017;45:1105–15. 28900017 10.1042/BST20160474

[btag090-B10] Calder PC. Omega‐3 polyunsaturated fatty acids and inflammatory processes: nutrition or pharmacology? Br J Clin Pharmacol 2013;75:645–62. 22765297 10.1111/j.1365-2125.2012.04374.x PMC3575932

[btag090-B11] Caponigro V , Tornesello AL, Merciai F et al Integrated plasma metabolomics and lipidomics profiling highlights distinctive signature of hepatocellular carcinoma in HCV patients. J Transl Med 2023;21:918. 38110968 10.1186/s12967-023-04801-4 PMC10729519

[btag090-B12] Chang T-Y , Li B-L, Chang CCY et al Acyl-coenzyme A: cholesterol acyltransferases. Am J Physiol Endocrinol Metab 2009;297:E1–E9. 19141679 10.1152/ajpendo.90926.2008 PMC2711667

[btag090-B13] Cho YK , Lee S, Lee J et al Lipid remodeling of adipose tissue in metabolic health and disease. Exp Mol Med 2023;55:1955–73. 37653032 10.1038/s12276-023-01071-4 PMC10545718

[btag090-B14] Conroy MJ , Andrews RM, Andrews S et al LIPID MAPS: update to databases and tools for the lipidomics community. Nucleic Acids Res 2024;52:D1677–D1682. 37855672 10.1093/nar/gkad896 PMC10767878

[btag090-B15] Czech MP , Tencerova M, Pedersen DJ et al Insulin signalling mechanisms for triacylglycerol storage. Diabetologia 2013;56:949–64. 23443243 10.1007/s00125-013-2869-1 PMC3652374

[btag090-B16] Dean JM , Lodhi IJ. Structural and functional roles of ether lipids. Protein Cell 2018;9:196–206. 28523433 10.1007/s13238-017-0423-5 PMC5818364

[btag090-B17] Dennis EA , Cao J, Hsu Y-H et al Phospholipase A_2_ enzymes: physical structure, biological function, disease implication, chemical inhibition, and therapeutic intervention. Chem Rev 2011;111:6130–85. 21910409 10.1021/cr200085w PMC3196595

[btag090-B18] Di Paolo G , De Camilli P. Phosphoinositides in cell regulation and membrane dynamics. Nature 2006;443:651–7. 17035995 10.1038/nature05185

[btag090-B19] Doll S , Proneth B, Tyurina YY et al ACSL4 dictates ferroptosis sensitivity by shaping cellular lipid composition. Nat Chem Biol 2017;13:91–8. 27842070 10.1038/nchembio.2239 PMC5610546

[btag090-B20] Escribá PV , Busquets X, Inokuchi J-I et al Membrane lipid therapy: modulation of the cell membrane composition and structure as a molecular base for drug discovery and new disease treatment. Prog Lipid Res 2015;59:38–53. 25969421 10.1016/j.plipres.2015.04.003

[btag090-B21] Fahy E , Subramaniam S, Brown HA et al A comprehensive classification system for lipids. J Lipid Res 2005;46:839–61. 15722563 10.1194/jlr.E400004-JLR200

[btag090-B22] Fahy E , Subramaniam S, Murphy RC et al Update of the LIPID MAPS comprehensive classification system for lipids. J Lipid Res 2009;50 Suppl:S9–14. 19098281 10.1194/jlr.R800095-JLR200 PMC2674711

[btag090-B23] Fajardo VA , McMeekin L, LeBlanc PJ et al Influence of phospholipid species on membrane fluidity: a meta-analysis for a novel phospholipid fluidity index. J Membr Biol 2011;244:97–103. 22052236 10.1007/s00232-011-9401-7

[btag090-B24] Farese RV , Walther TC. Lipid droplets finally get a little R-E-S-P-E-C-T. Cell 2009;139:855–60. 19945371 10.1016/j.cell.2009.11.005 PMC3097139

[btag090-B25] Farooqui AA. Studies on plasmalogen-selective phospholipase A2 in brain. Mol Neurobiol 2010;41:267–73. 20049656 10.1007/s12035-009-8091-y

[btag090-B26] Fontaine D , Figiel S, Félix R et al Roles of endogenous ether lipids and associated PUFAs in the regulation of ion channels and their relevance for disease. J Lipid Res 2020;61:840–58. 32265321 10.1194/jlr.RA120000634 PMC7269763

[btag090-B27] Foster DA , Salloum D, Menon D et al Phospholipase D and the maintenance of phosphatidic acid levels for regulation of mammalian target of rapamycin (mTOR). J Biol Chem 2014;289:22583–8. 24990952 10.1074/jbc.R114.566091 PMC4132766

[btag090-B28] Fruhwirth GO , Loidl A, Hermetter A et al Oxidized phospholipids: from molecular properties to disease. Biochim Biophys Acta 2007;1772:718–36. 17570293 10.1016/j.bbadis.2007.04.009

[btag090-B29] Gaud C , C Sousa B, Nguyen A et al BioPAN: a web-based tool to explore mammalian lipidome metabolic pathways on LIPID MAPS. F1000Res 2021;10:4. 33564392 10.12688/f1000research.28022.1 PMC7848852

[btag090-B30] Gimeno RE , Cao J. Thematic review series: glycerolipids. Mammalian glycerol-3-phosphate acyltransferases: new genes for an old activity. J Lipid Res 2008;49:2079–88. 18658143 10.1194/jlr.R800013-JLR200

[btag090-B31] Grevengoed TJ , Klett EL, Coleman RA et al Acyl-CoA metabolism and partitioning. Annu Rev Nutr 2014;34:1–30. 24819326 10.1146/annurev-nutr-071813-105541 PMC5881898

[btag090-B32] Gurr MI , Harwood JL, Frayn KN, et al Lipids: Biochemistry, Biotechnology and Health. 6th edn. Chichester: Wiley-Blackwell, 2016.

[btag090-B33] Hannun YA , Obeid LM. Sphingolipids and their metabolism in physiology and disease. Nat Rev Mol Cell Biol 2018;19:175–91. 29165427 10.1038/nrm.2017.107 PMC5902181

[btag090-B34] Harayama T , Riezman H. Understanding the diversity of membrane lipid composition. Nat Rev Mol Cell Biol 2018;19:281–96. 29410529 10.1038/nrm.2017.138

[btag090-B35] Henze E , Burkhardt RN, Fox BW, et al 2025. ATP-release pannexin channels are gated by lysophospholipids. bioRxiv. 10.23.563601, 2023. 10.7554/eLife.107067 PMC12045621 40309905

[btag090-B36] Hisano K , Kawase S, Mimura T et al Structurally different lysophosphatidylethanolamine species stimulate neurite outgrowth in cultured cortical neurons via distinct G-protein-coupled receptors and signaling cascades. Biochem Biophys Res Commun 2021;534:179–85. 33298313 10.1016/j.bbrc.2020.11.119

[btag090-B37] Hornberger TA , Chu WK, Mak YW et al The role of phospholipase D and phosphatidic acid in the mechanical activation of mTOR signaling in skeletal muscle. Proc Natl Acad Sci USA 2006;103:4741–6. 16537399 10.1073/pnas.0600678103 PMC1450240

[btag090-B38] Hornemann T. Lipidomics in biomarker research. Handb Exp Pharmacol 2022;270:493–510. 34409495 10.1007/164_2021_517

[btag090-B39] Hullin-Matsuda F , Luquain-Costaz C, Bouvier J et al Bis(monoacylglycero)phosphate, a peculiar phospholipid to control the fate of cholesterol: implications in pathology. Prostaglandins Leukot Essent Fatty Acids 2009;81:313–24. 19857945 10.1016/j.plefa.2009.09.006

[btag090-B40] Itri R , Junqueira HC, Mertins O et al Membrane changes under oxidative stress: the impact of oxidized lipids. Biophys Rev 2014;6:47–61. 28509959 10.1007/s12551-013-0128-9 PMC5425709

[btag090-B41] Kagan VE , Jiang J, Huang Z et al NDPK-D (NM23-H4)-mediated externalization of cardiolipin enables elimination of depolarized mitochondria by mitophagy. Cell Death Differ 2016;23:1140–51. 26742431 10.1038/cdd.2015.160 PMC4946882

[btag090-B42] Lass A , Zimmermann R, Oberer M et al Lipolysis – a highly regulated multi-enzyme complex mediates the catabolism of cellular fat stores. Prog Lipid Res 2011;50:14–27. 21087632 10.1016/j.plipres.2010.10.004 PMC3031774

[btag090-B43] Law S-H , Chan M-L, Marathe GK et al An updated review of lysophosphatidylcholine metabolism in human diseases. IJMS 2019;20:1149. 30845751 10.3390/ijms20051149 PMC6429061

[btag090-B44] Lee J-Y , Kim WK, Bae K-H et al Lipid metabolism and ferroptosis. Biology (Basel) 2021;10:184. 33801564 10.3390/biology10030184 PMC8000263

[btag090-B45] Lee KS , Su X, Huan T et al Metabolites are not genes–avoiding the misuse of pathway analysis in metabolomics. Nat Metab 2025;7:858–61. 40211046 10.1038/s42255-025-01283-0

[btag090-B46] Li Z , Agellon LB, Allen TM et al The ratio of phosphatidylcholine to phosphatidylethanolamine influences membrane integrity and steatohepatitis. Cell Metab 2006;3:321–31. 16679290 10.1016/j.cmet.2006.03.007

[btag090-B47] Lin DA , Boyce JA. Lysophospholipids as mediators of immunity. Adv Immunol 2006;89:141–67. 16682274 10.1016/S0065-2776(05)89004-2

[btag090-B48] Lin W-J , Shen P-C, Liu H-C et al LipidSig: a web-based tool for lipidomic data analysis. Nucleic Acids Res 2021;49:W336–W345. 34048582 10.1093/nar/gkab419 PMC8262718

[btag090-B49] Lingwood D , Simons K. Lipid rafts as a membrane-organizing principle. Science 2010;327:46–50. 20044567 10.1126/science.1174621

[btag090-B50] Listenberger LL , Han X, Lewis SE et al Triglyceride accumulation protects against fatty acid-induced lipotoxicity. Proc Natl Acad Sci USA 2003;100:3077–82. 12629214 10.1073/pnas.0630588100 PMC152249

[btag090-B51] Liu L , Ye Y, Lin R et al Ferroptosis: a promising candidate for exosome-mediated regulation in different diseases. Cell Commun Signal 2024;22:6. 38166927 10.1186/s12964-023-01369-w PMC11057189

[btag090-B52] Lu Y , Pang Z, Xia J et al Comprehensive investigation of pathway enrichment methods for functional interpretation of LC–MS global metabolomics data. Brief Bioinform 2023;24:bbac553. 36572652 10.1093/bib/bbac553 PMC9851290

[btag090-B53] Maxfield FR , Tabas I. Role of cholesterol and lipid organization in disease. Nature 2005;438:612–21. 16319881 10.1038/nature04399

[btag090-B54] Mett J , Lauer AA, Janitschke D et al Medium-chain length fatty acids enhance Aβ degradation by affecting insulin-degrading enzyme. Cells 2021;10:2941. 34831163 10.3390/cells10112941 PMC8616162

[btag090-B55] Meyer Zu Heringdorf D , Jakobs KH. Lysophospholipid receptors: signalling, pharmacology and regulation by lysophospholipid metabolism. Biochim Biophys Acta 2007;1768:923–40. 17078925 10.1016/j.bbamem.2006.09.026

[btag090-B56] Moessinger C , Klizaite K, Steinhagen A et al Two different pathways of phosphatidylcholine synthesis, the kennedy pathway and the lands cycle, differentially regulate cellular triacylglycerol storage. BMC Cell Biol 2014;15:43. 25491198 10.1186/s12860-014-0043-3 PMC4293825

[btag090-B57] Molenaar MR , Jeucken A, Wassenaar TA et al LION/web: a web-based ontology enrichment tool for lipidomic data analysis. Gigascience 2019;8:giz061. 31141612 10.1093/gigascience/giz061 PMC6541037

[btag090-B58] Murakami M , Sato H, Taketomi Y et al Updating phospholipase A2 biology. Biomolecules 2020;10:1457. 33086624 10.3390/biom10101457 PMC7603386

[btag090-B59] Nakamura MT , Yudell BE, Loor JJ et al Regulation of energy metabolism by long-chain fatty acids. Prog Lipid Res 2014;53:124–44. 24362249 10.1016/j.plipres.2013.12.001

[btag090-B60] Newton AC. Protein kinase C: poised to signal. Am J Physiol Endocrinol Metab 2010;298:E395–E402. 19934406 10.1152/ajpendo.00477.2009 PMC2838521

[btag090-B61] Nguyen A , Rudge SA, Zhang Q et al Using lipidomics analysis to determine signalling and metabolic changes in cells. Curr Opin Biotechnol 2017;43:96–103. 27816901 10.1016/j.copbio.2016.10.003

[btag090-B62] Ntambi JM. Regulation of stearoyl-CoA desaturase by polyunsaturated fatty acids and cholesterol. J Lipid Res 1999;40:1549–58. 10484602

[btag090-B63] Ogretmen B. Sphingolipid metabolism in cancer signalling and therapy. Nat Rev Cancer 2018;18:33–50. 29147025 10.1038/nrc.2017.96 PMC5818153

[btag090-B64] Papamandjaris AA , MacDougall DE, Jones PJ et al Medium chain fatty acid metabolism and energy expenditure: obesity treatment implications. Life Sci 1998;62:1203–15. 9570335 10.1016/s0024-3205(97)01143-0

[btag090-B65] Paradies G , Paradies V, Ruggiero FM et al Role of cardiolipin in mitochondrial function and dynamics in health and disease: molecular and pharmacological aspects. Cells 2019;8:728. 31315173 10.3390/cells8070728 PMC6678812

[btag090-B66] Pellegrino RM , Giulietti M, Alabed HBR et al LipidOne: user-friendly lipidomic data analysis tool for a deeper interpretation in a systems biology scenario. Bioinformatics 2022;38:1767–9. 34971364 10.1093/bioinformatics/btab867

[btag090-B67] Prokazova NV , Zvezdina ND, Korotaeva AA et al Effect of lysophosphatidylcholine on transmembrane signal transduction. Biochemistry (Mosc) 1998;63:31–7. 9526092

[btag090-B68] Ramstedt B , Slotte JP. Interaction of cholesterol with sphingomyelins and Acyl-Chain-Matched phosphatidylcholines: a comparative study of the effect of the chain length. Biophys J 1999;76:908–15. 9929492 10.1016/S0006-3495(99)77254-1 PMC1300092

[btag090-B69] Rangholia N , Leisner TM, Holly SP et al Bioactive ether lipids: primordial modulators of cellular signaling. Metabolites 2021;11:41. 33430006 10.3390/metabo11010041 PMC7827237

[btag090-B70] Reue K , Brindley DN. Thematic review series: glycerolipids. Multiple roles for lipins/phosphatidate phosphatase enzymes in lipid metabolism. J Lipid Res 2008;49:2493–503. 18791037 10.1194/jlr.R800019-JLR200 PMC2582367

[btag090-B71] Róg T , Murzyn K, Gurbiel R et al Effects of phospholipid unsaturation on the bilayer nonpolar region: a molecular simulation study. J Lipid Res 2004;45:326–36. 14594994 10.1194/jlr.M300187-JLR200

[btag090-B72] Sachinidis A , Kraus R, Seul C et al Gangliosides GM1, GM2 and GM3 inhibit the platelet-derived growth factor-induced signalling transduction pathway in vascular smooth muscle cells by different mechanisms. Eur J Cell Biol 1996;71:79–88. 8884181

[btag090-B73] Samra JS. Regulation of lipid metabolism in adipose tissue. Proc Nutr Soc 2000;59:441–6. 10997671 10.1017/s0029665100000604

[btag090-B74] Schönfeld P , Wojtczak L. Short- and medium-chain fatty acids in energy metabolism: the cellular perspective. J Lipid Res 2016;57:943–54. 27080715 10.1194/jlr.R067629 PMC4878196

[btag090-B75] Serhan CN , Chiang N, Van Dyke TE et al Resolving inflammation: dual anti-inflammatory and pro-resolution lipid mediators. Nat Rev Immunol 2008;8:349–61. 18437155 10.1038/nri2294 PMC2744593

[btag090-B76] Shao Y , Nanayakkara G, Cheng J et al Lysophospholipids and their receptors serve as conditional DAMPs and DAMP receptors in tissue oxidative and inflammatory injury. Antioxid Redox Signal 2018;28:973–86. 28325059 10.1089/ars.2017.7069 PMC5849278

[btag090-B77] Shevchenko A , Simons K. Lipidomics: coming to grips with lipid diversity. Nat Rev Mol Cell Biol 2010;11:593–8. 20606693 10.1038/nrm2934

[btag090-B78] Simons K , Ikonen E. Functional rafts in cell membranes. Nature 1997;387:569–72. 9177342 10.1038/42408

[btag090-B79] Simopoulos AP. The importance of the omega-6/omega-3 fatty acid ratio in cardiovascular disease and other chronic diseases. Exp Biol Med (Maywood) 2008;233:674–88. 18408140 10.3181/0711-MR-311

[btag090-B80] Slotte JP. Sphingomyelin–cholesterol interactions in biological and model membranes. Chem Phys Lipids 1999;102:13–27. 11001557 10.1016/s0009-3084(99)00071-7

[btag090-B81] Stillwell W , Wassall SR. Docosahexaenoic acid: membrane properties of a unique fatty acid. Chem Phys Lipids 2003;126:1–27. 14580707 10.1016/s0009-3084(03)00101-4

[btag090-B82] Stith JL , Velazquez FN, Obeid LM et al Advances in determining signaling mechanisms of ceramide and role in disease. J Lipid Res 2019;60:913–8. 30846529 10.1194/jlr.S092874 PMC6495170

[btag090-B83] Subczynski WK , Pasenkiewicz-Gierula M, Widomska J et al High cholesterol/low cholesterol: effects in biological membranes: a review. Cell Biochem Biophys 2017;75:369–85. 28417231 10.1007/s12013-017-0792-7 PMC5645210

[btag090-B84] Sych T , Levental KR, Sezgin E et al Lipid-Protein interactions in plasma membrane organization and function. Annu Rev Biophys 2022;51:135–56. 34982570 10.1146/annurev-biophys-090721-072718 PMC12101515

[btag090-B85] Tajima O , Fujita Y, Ohmi Y et al Ganglioside GM3 prevents high fat diet-induced hepatosteatosis via attenuated insulin signaling pathway. PLoS One 2023;18:e0281414. 36827398 10.1371/journal.pone.0281414 PMC9956598

[btag090-B86] Thakkar H , Vincent V, Chaurasia B et al Ceramide signaling in immunity: a molecular perspective. Lipids Health Dis 2025;24:225. 40597360 10.1186/s12944-025-02642-2 PMC12218095

[btag090-B87] Uzman A. Molecular cell biology (4th edition) Harvey Lodish, Arnold Berk, S. Lawrence Zipursky, Paul Matsudaira, David Baltimore and James Darnell; Freeman & Co., New York, NY, 2000, 1084 pp., list price $102.25, ISBN 0-7167-3136-3. Biochem Mol Biol Educ 2001;29:126–8.

[btag090-B88] van Meer G , Voelker DR, Feigenson GW et al Membrane lipids: where they are and how they behave. Nat Rev Mol Cell Biol 2008;9:112–24. 18216768 10.1038/nrm2330 PMC2642958

[btag090-B89] Walther TC , Farese RV. Lipid droplets and cellular lipid metabolism. Annu Rev Biochem 2012;81:687–714. 22524315 10.1146/annurev-biochem-061009-102430 PMC3767414

[btag090-B90] Weiss-Hersh K , Garcia AL, Marosvölgyi T et al Saturated and monounsaturated fatty acids in membranes are determined by the gene expression of their metabolizing enzymes SCD1 and ELOVL6 regulated by the intake of dietary fat. Eur J Nutr 2020;59:2759–69. 31676951 10.1007/s00394-019-02121-2 PMC7413877

[btag090-B91] Werner ER , Keller MA, Sailer S et al The *TMEM189* gene encodes plasmanylethanolamine desaturase which introduces the characteristic vinyl ether double bond into plasmalogens. Proc Natl Acad Sci USA 2020;117:7792–8. 32209662 10.1073/pnas.1917461117 PMC7149458

[btag090-B92] Yaginuma S , Omi J, Kano K et al Lysophospholipids and their producing enzymes: their pathological roles and potential as pathological biomarkers. Pharmacol Ther 2023;246:108415. 37061204 10.1016/j.pharmthera.2023.108415

[btag090-B93] Yamamoto Y , Sakurai T, Chen Z et al Lysophosphatidylethanolamine affects lipid accumulation and metabolism in a human liver-derived cell line. Nutrients 2022;14:579. 35276938 10.3390/nu14030579 PMC8839386

[btag090-B94] Yang HC , Farooqui AA, Horrocks LA et al Plasmalogen-selective phospholipase A2 and its role in signal transduction. J Lipid Mediat Cell Signal 1996;14:9–13. 8906539 10.1016/0929-7855(96)01502-7

[btag090-B95] Yen C-LE , Stone SJ, Koliwad S et al Thematic review series: glycerolipids. DGAT enzymes and triacylglycerol biosynthesis. J Lipid Res 2008;49:2283–301. 18757836 10.1194/jlr.R800018-JLR200 PMC3837458

[btag090-B96] Zimmermann R , Strauss JG, Haemmerle G et al Fat mobilization in adipose tissue is promoted by adipose triglyceride lipase. Science 2004;306:1383–6. 15550674 10.1126/science.1100747

