# Supplementary material for: Functional lipid analysis via index-based lipidomics profile: a new computational module in LipidOne
Source: Bioinformatics. 2026 Mar 1;42(3):btag090. doi: 10.1093/bioinformatics/btag090 (PMC12970596; doi:10.1093/bioinformatics/btag090)
Supplement: btag090_Supplementary_Data [file btag090_supplementary_data.zip › Supplemental_HA.docx]

**Supplemental of**

Functional Lipid Analysis via Index-Based Lipidomics Profile: A New Computational Module in LipidOne

**Authors**: Alabed, H. B. R.^1*^, Mancini, D. F.^2^, Pergola M.^2^, Romani, L. ^3^, Martino S. ^2^, Koulman A.^1^, Pellegrino, R. M.^2^

^1^ Institute of Metabolic Science-Metabolic Research Laboratories, University of Cambridge, Cambridge, UK.

^2^ University of Perugia, Dept. Chemistry, Biology and Biotechnology, Via del Giochetto, building B, 06126 - Perugia – Italy

^3^ San Raffaele Research Center, 67039 Sulmona, L’Aquila, Italy

^*^ To whom correspondence should be addressed.

Contact: ha581@cam.ac.uk

**Case studies using Functional Lipid Analysis**

Mechanistic interpretations in this section should be read as FLA-inferred hypotheses, derived from directional changes in lipid indices and prior biochemical knowledge, rather than direct measurements of enzyme activity or organelle remodeling.

**Functional Lipid Analysis captures impaired lipid utilization upon Pyruvate Dehydrogenase Kinase-1 knockdown in** **cardiomyocytes**

In this study, the authors reported that Pyruvate Dehydrogenase Kinase-1 (PDK1) knockdown (KD) led to impaired lipid mobilization and reduced energy buffering compared to scrambled controls (SC), as evidenced by changes in lipid classes and metabolic markers. They observed a shift towards enhanced lipid storage and reduced fatty acid oxidation, indicative of a metabolic imbalance.

To explore the global lipid functional landscape in response to PDK1 knockdown, we performed principal component analysis (PCA) using the set of functional lipid indices computed by the FLA module.


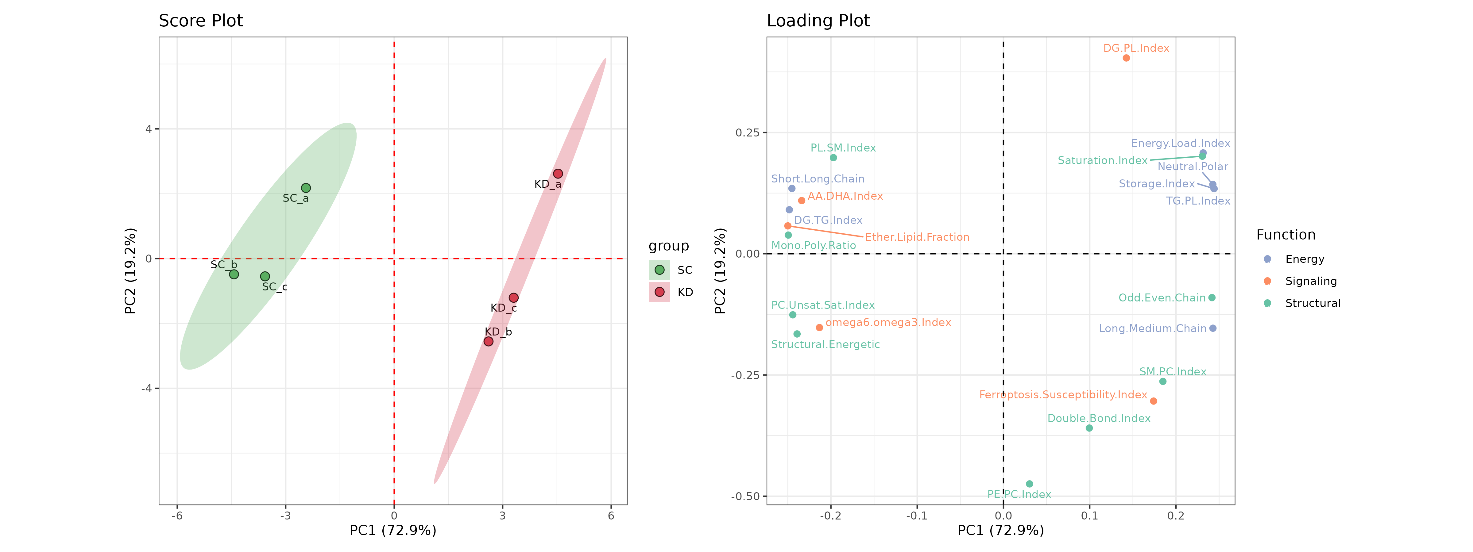


Figure S1: Principal component analysis of functional lipid indices in PDK1 knockdown (KD) versus control (SC) cardiomyocytes: Left: The score plot shows a complete separation between KD and SC samples along PC1 (72.9% of variance), with minimal intra-group variability. Right: The loading plot indicates that energy-related indices (e.g., Storage Index, TG/PL Index, Energy Load Index) strongly contribute to the first principal component, in agreement with the lipid functional shift observed in KD cells. Structural and signaling indices show more modest contributions along PC2 (19.2%), confirming that the dominant functional alteration lies in energy lipid metabolism.

The score plot revealed a clear separation between KD and SC along PC1 (72.9% of variance), capturing the dominant functional difference in the dataset. The loading plot confirmed that energy-related indices—Storage Index, TG/PL Index, Energy Load Index—were the main contributors, aligning with the KD condition. In contrast, structural and signaling indices contributed more modestly along PC2, indicating that the primary alteration is related to energetic lipid turnover.

**Functional Summary and Lipid Domain** **Shifts**

Using the Functional Summary feature, we compared all indices between KD and SC cells under nutrient deprivation. This analysis revealed a selective shift across the three major lipid domains—structural, signaling, and energy.


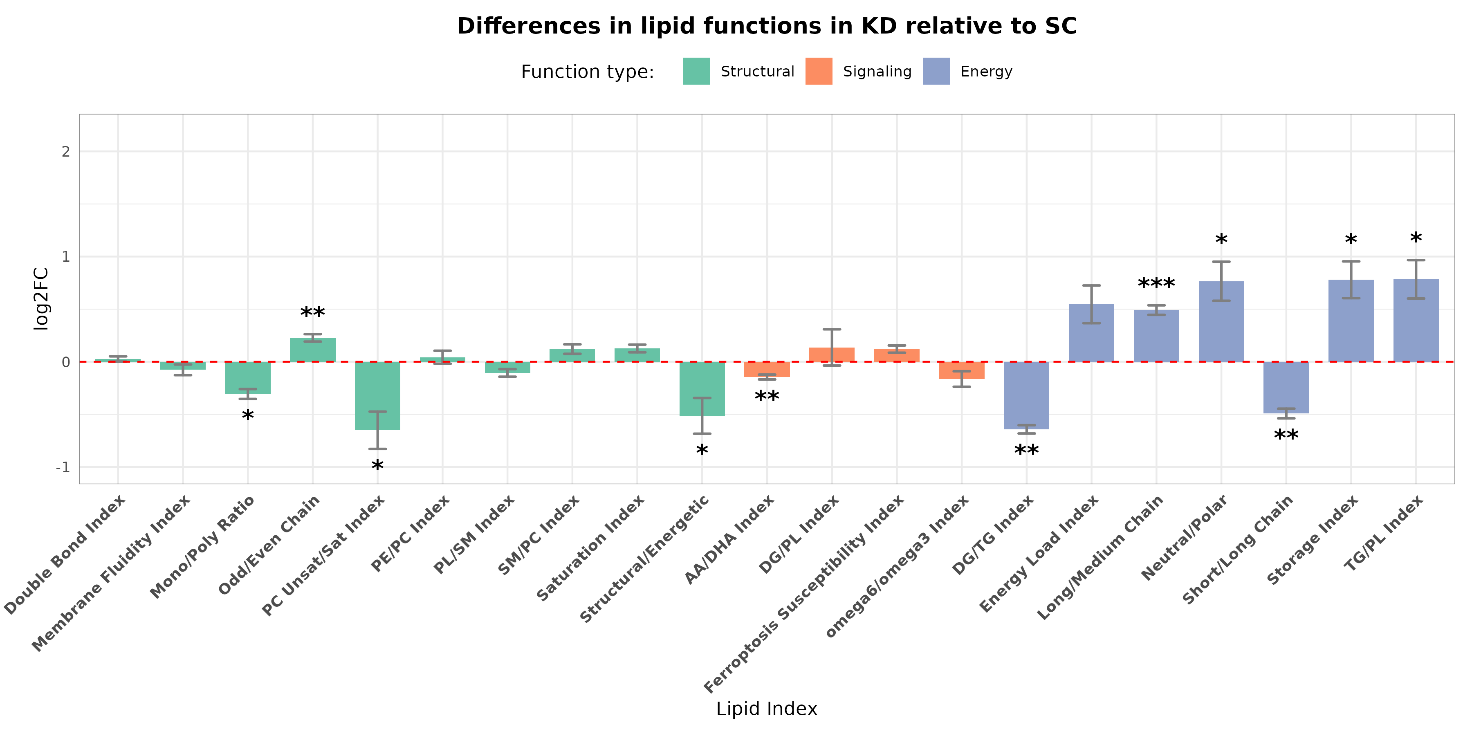


Figure S2: Differences in functional lipid indices in PDK1 knockdown (KD) versus scrambled control (SC) cardiomyocytes under nutrient deprivation. Bar plot showing log₂ fold-change (log₂FC) values for functional lipid indices calculated by the FLA module, grouped by lipid function: structural (green), signaling (orange), and energy (blue). Positive values indicate higher index values in KD, negative values indicate lower values relative to SC. KD cells exhibited a pronounced upregulation of storage-related energy indices (e.g., Storage Index, TG/PL Index, Long/Medium Chain), alongside significant downregulation of mobilization-related indices (Energy Index, DG/TG Index), indicative of enhanced lipid storage but reduced lipolytic flux and energy release capacity. Selected signaling indices (Ether Lipid Fraction, AA/DHA Index) were also significantly decreased, suggesting altered lipid-mediated signaling and potential anti-inflammatory adaptation. Asterisks denote statistical significance (*p < 0.05; **p < 0.01; ***p < 0.001).

Notably, while structural lipids exhibited modest and heterogeneous changes, energy-related lipid indices were strongly upregulated in KD. The Storage Index, TG/PL Index, and Energy Load Index showed significantly positive log2 fold-changes (Figure S2), consistent with the experimentally observed impairment in triacylglycerol hydrolysis. By contrast, the DG/TG Index was significantly decreased, consistent with a relative accumulation of triacylglycerols over diacylglycerols and a reduced lipolytic flux. Together with the increases in the Storage Index, TG/PL Index and Energy Load Index, this pattern indicates enhanced neutral-lipid storage but a diminished capacity to mobilize stored lipids into readily oxidizable substrates (significance as indicated in Figure S2).

Interestingly, certain signaling lipid indices—such as the Ether Lipid Fraction and AA/DHA Index—were significantly downregulated. This suggests diminished ether lipid synthesis or turnover, potentially altering response to oxidative stress (Jové *et al.*, 2023; Perez *et al.*, 2022) and changing specific lipid-mediated cellular pathways (Dean and Lodhi, 2018). The reduced AA/DHA ratio could suggest an alteration in anti or/and anti - inflammatory processes. Such findings highlight the added value of a function-based approach, which can uncover subtle, yet biologically relevant shifts often missed in compositional or class-based analyses.

**Molecular mechanisms and protein-lipid interaction network**


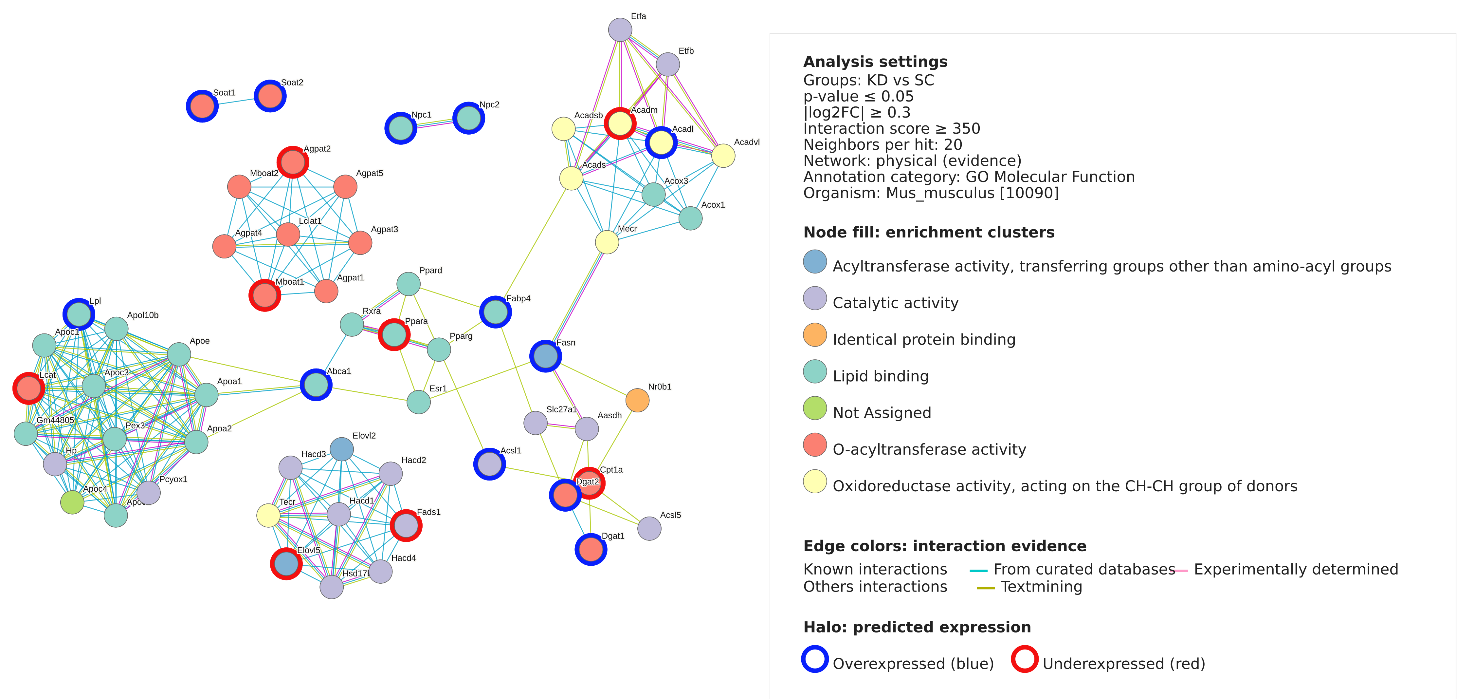


Figure S3: FLA-guided lipid–protein interaction network in mouse cardiomyocytes after PDK1 knockdown. Nodes are mouse protein symbols; edges are STRING functional associations (edge color = evidence channel). Node fill = GO Molecular Function enrichment clusters. Node halos = FLA-predicted expression status (blue, overexpressed; red, under-expressed). The layout highlights four major regions: FAO/β-oxidation (left), lipid-binding/lipoprotein cluster (upper), acyl-chain editing (right, e.g., Fasn, Scd1, Elovl5/6, Fads1/2), and acyltransferases/glycerolipid remodeling (lower-right, e.g., Agpat, Lclat1, Lpcat, together with Lpin/Dgat). This organization aligns with FLA’s functional readouts indicating increased storage alongside reduced fatty-acid utilization in KD.

To probe mechanism, we built an FLA-guided STRING network (mouse protein symbols). Node fill encodes GO Molecular Function enrichment clusters, edge colors show STRING evidence channels, and node halos report the FLA-predicted direction (blue, overexpressed; red, under-expressed). The layout reveals four coherent modules:

FAO/β-oxidation. Enzymes for activation, import and mitochondrial β-oxidation (e.g., Acsl, Cpt1/2, Acadvl/Acadm, Eci1, Decr1, Etfa/b, Hadha/b, Acaa1/2). The mixed halo pattern, with several under-expressed nodes, is consistent with the FLA inference of lower mitochondrial FA utilization in KD.

**Lipid-binding/lipoprotein cluster**. A densely connected group of lipid-binding proteins and apolipoproteins, indicating changes in lipid handling and trafficking at the interface with extracellular/particle-associated pathways.

**Acyl-chain editing**. De novo synthesis, elongation and desaturation (e.g., Fasn, Scd1, Elovl5/6, Fads1/2, Hsd17b12), consistent with adjustments in chain length and unsaturation that accompany the storage-dominant phenotype.

**Acyltransferases/glycerolipid remodelling**. Lysophospholipid and glycerolipid acyltransferases (e.g., Agpat family, Lclat1, Lpcat), together with phosphatidate/diacylglycerol branch enzymes (e.g., Lpin, Dgat), supporting enhanced neutral-lipid storage and ongoing phospholipid remodeling.

Across modules, the network maps FLA-inferred functional shifts onto enzyme-level hypotheses (e.g., the Pnpla2/ATGL axis and the FAO machinery), providing a mechanistic scaffold for follow-up experiments.

The FLA results agree with the original study showing that PDK1 knockdown impairs lipid mobilization and fatty-acid oxidation (FAO). Beyond this confirmation, FLA resolves additional features: indices indicate concurrent lipid storage increase with reduced mobilization (e.g., Energy Index, DG/TG), consistent with a lower lipolytic flux and limited rapid energy release. It also detects shifts in signaling-lipid indices and predicts coherent changes in the protein network, meriting follow-up.

In the same dataset, FLA recapitulates lipid signatures consistent with reduced TAG mobilization, the reduced mitochondrial FAO capacity, and the raises the hypothesis of decreased ATGL-linked lipolysis in KD cells. Moreover, it captures functional shifts that parallel the subtle remodeling of TG acyl-chain length and unsaturation reported by the authors—patterns that bulk compositional views can miss. Thus, FLA reproduces the study’s in vitro/in vivo convergence directly from lipidomics, offering a rapid, mechanism-oriented readout of the phenotype.

Finally, FLA adds mechanistic resolution by quantifying the divergence between storage-positive (Storage, TG/PL, Energy Load) and mobilization-negative (Energy, DG/TG) indices; by revealing organelle-aware changes (elevated LPE/PE consistent with increased PE remodeling and suggestive of inner-mitochondrial membrane dynamics, together with reduced ether-lipid and AA/DHA signals); and by mapping these alterations onto a Pdk1–Pdk4–Pdha1-centred network connected to Pnpla2 (ATGL) and the FAO machinery. Together, these results show that a single lipidomics dataset can yield a function-based interpretation that confirms impaired lipid utilization and prioritizes enzyme-level, testable hypotheses underlying the cardiomyocyte phenotype—within minutes.

Functional Lipid Analysis of lipidomic signatures in Hepatocellular carcinoma and chronic hepatitis C virus -related conditions

Hepatocellular carcinoma (HCC) is the most common primary liver malignancy, often arising in patients with chronic hepatitis C virus (HCV) infection. Early diagnosis remains a clinical challenge, particularly in AFP-negative patients, where traditional biomarkers and imaging approaches show limited sensitivity. In a recent multi-omics investigation, Caponigro et al. (2023) (Caponigro *et al.*, 2023) performed integrated untargeted metabolomics and lipidomics profiling of plasma from 102 HCV-positive subjects, including patients with HCC (n = 69), chronic HCV infection without cancer (n = 23), and HCV-associated mixed cryoglobulinemia (MC, n = 10). Using HILIC-HRMS for polar metabolites and RP-UHPLC-HRMS for lipids, the authors identified distinctive metabolic and lipidomic signatures for HCC. Key findings included: Marked elevation of short- and long-chain acylcarnitines in HCC, consistent with altered mitochondrial β-oxidation; Pronounced reduction of lysophosphatidylcholines (LPCs), both saturated and unsaturated, suggesting dysregulation of phospholipid remodeling via the Lands’ cycle; Pathway enrichment highlighting mitochondrial β-oxidation of short-chain saturated fatty acids and phospholipid biosynthesis as significantly modulated in HCC; Supervised modeling (PLS-DA) demonstrating that combined metabolomics and lipidomics datasets outperform AFP for distinguishing HCC from HCV and MC, including AFP-negative cases, with AUC values up to 0.94.

Here, we re-analysed the publicly available lipidomics dataset from this study using the Functional Lipid Analysis (FLA) module of LipidOne.

Functional alterations in HCC versus HCV chronic infection

To obtain an overview of functional lipid differences between HCC and chronic HCV infection, we inspected the bubble heatmap of the most perturbed FLA indices (Figure S4). Color encodes the log₂ fold change for HCV relative to HCC (red = higher in HCV; blue = lower), and bubble size scales with statistical support (−log₁₀ p-value). Each of the 10 main indices is accompanied by a concise sentence that facilitates its functional interpretation.


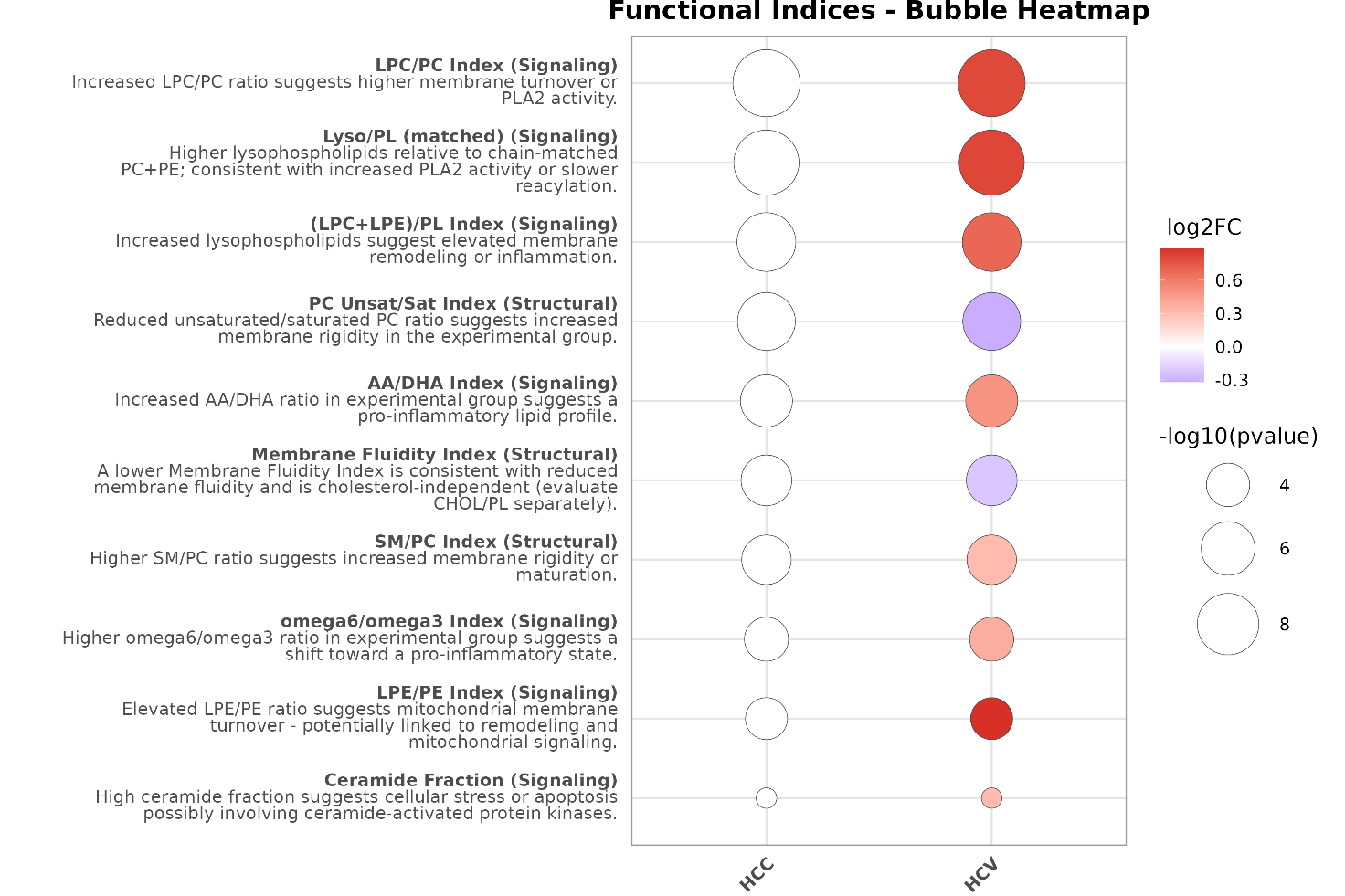


*Figure S4: Functional lipid indices distinguishing chronic HCV infection from HCC. Bubble heatmap of selected FLA indices comparing HCV vs HCC. Bubble color shows log₂(HCV/HCC) (red = higher in HCV; blue = lower), and size is proportional to −log₁₀(p-value). The strongest differences are driven by lysophospholipid-related indices and by LPE/PE, while PC Unsat/Sat and the Membrane Fluidity Index trend lower in* *HCV*.

A clear predominance of signaling-related alterations emerged. HCV showed robust increases in multiple lysophospholipid indices—LPC/PC, (LPC+LPE)/PL, and Lyso/PL (matched)—consistent with enhanced PLA2 activity and/or slower reacylation compared with HCC. The LPE/PE index was also markedly elevated in HCV, suggesting increased turnover of inner/mitochondrial membranes.

In contrast, PC Unsat/Sat and the Membrane Fluidity Index decreased in HCV, indicating reduced membrane fluidity (greater rigidity); SM/PC was modestly higher in HCV, pointing in the same direction of increased membrane order. Pro-inflammatory balance indices (AA/DHA and omega-6/omega-3) were higher in HCV, consistent with a more pro-inflammatory lipid milieu. The Ceramide Fraction was slightly lower in HCV, arguing against enhanced ceramide-driven stress relative to HCC. Altogether, these patterns recapitulate the lysophospholipid depletion reported in HCC by Caponigro et al., while translating lipid changes into a functional framework linking membrane remodeling, inflammation, and organelle turnover.

**Identification of the most discriminant functional indices between HCC and** **HCV**

The functional volcano plot (Figure S5) summarizes effect size [log2(HCV/HCC)] versus significance (−log10 p) across Energy, Signaling, and Structural indices. Consistent with Caponigro et al., we confirm a marked depletion of lysophospholipids in HCC: indices that rise in HCV include LPC/PC, (LPC+LPE)/PL, and Lyso/PL (matched), all exceeding both effect-size and significance thresholds. FLA adds two functional refinements not apparent from class-level lipid lists alone. First, the ether-specific Lyso-O/PL-O (matched) index is also higher in HCV, indicating that PLA2-linked turnover extends to ether lipids, a detail that implicates peroxisomal/ER pathways of membrane remodeling. Second, LPE/PE is strongly increased in HCV, pointing to heightened inner membrane/mitochondrial remodeling relative to HCC.


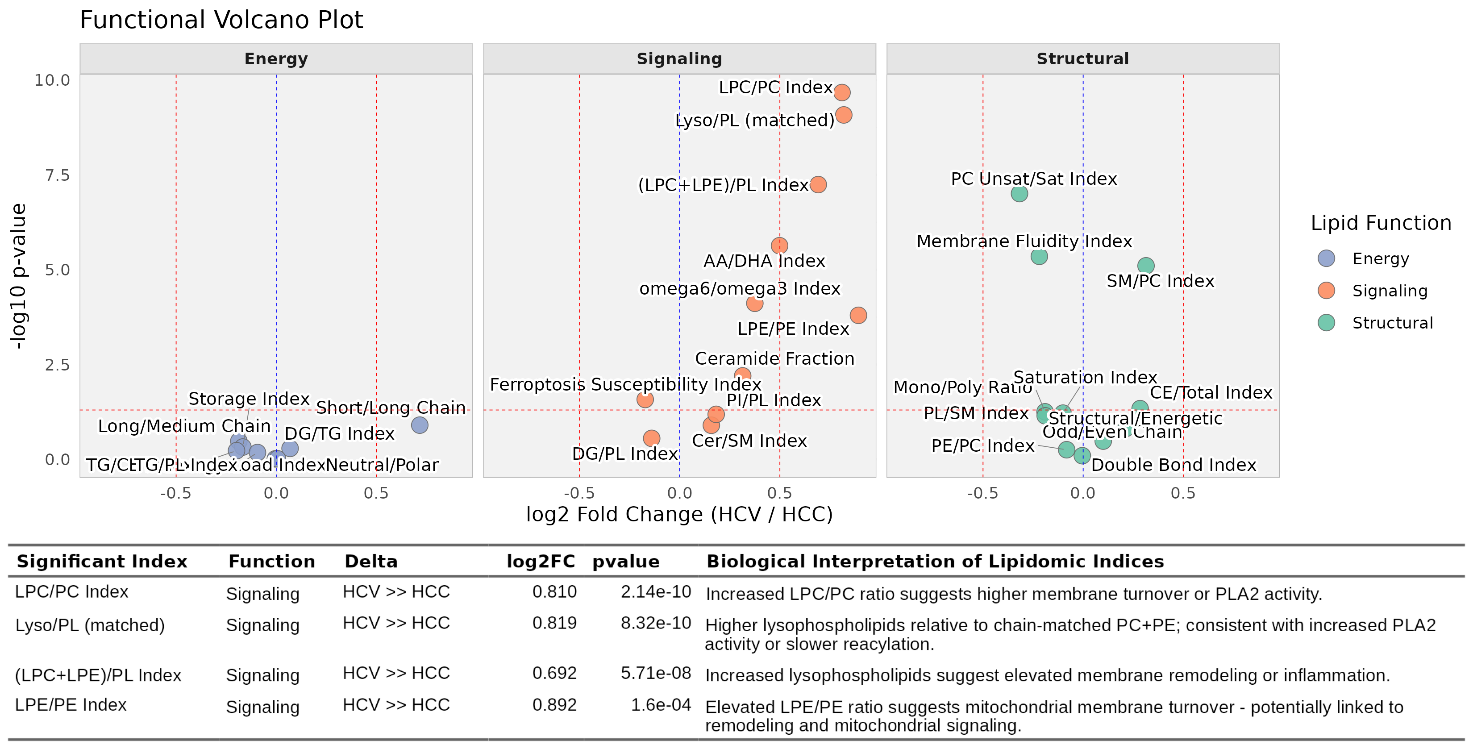


Figure S5: Functional Volcano Plot (HCV vs HCC). Each point is an FLA index plotted by log₂(HCV/HCC) (x-axis) and −log₁₀(p-value) (y-axis), grouped by lipid function (Energy, Signaling, Structural). Vertical dashed lines mark the user-defined fold-change band; the horizontal dashed line marks p = 0.05. Labels indicate indices exceeding both thresholds; the table reports these hits with direction and statistics. The significant hits are dominated by signaling/structural indices, whereas energy-related indices remain near the origin.

Among signaling ratios, HCV displays four large-effect, significant increases—LPC/PC, (LPC+LPE)/PL, Lyso/PL (matched), and LPE/PE— consistent with PLA2-linked processes / suggests increased diacylation and/or slower reacylation together with increased turnover of inner/mitochondrial membranes. Pro-inflammatory balance indices (AA/DHA, omega-6/omega-3) tend to be higher in HCV but fall inside the preset effect-size band. The Ceramide Fraction shows only a modest, non-significant shift, providing no evidence for stronger ceramide-driven signaling in either group.

Structural trends include lower PC Unsat/Sat and a lower Membrane Fluidity Index, together with slightly higher SM/PC in HCV—patterns consistent with more ordered (less fluid) membranes—yet these remain below the combined thresholds. Energy-related indices cluster near the origin and do not pass the filters, indicating that the dominant differences between HCV and HCC are signaling/structural rather than storage-centric. Overall, the volcano plot corroborates the depletion of lysophospholipids in HCC reported by Caponigro et al. and reframes the lipid changes in a functional context that links membrane remodeling, inflammation, and organelle turnover.

Functional biomarker discovery for HCC detection in HCV-positive patients

From the complete panel of Functional Lipid Analysis (FLA) indices, we selected the top 20 discriminators for HCV vs HCC (Figure S6). For each index we report p-value, ROC AUC, statistical power, and Cohen’s d to quantify diagnostic potential.


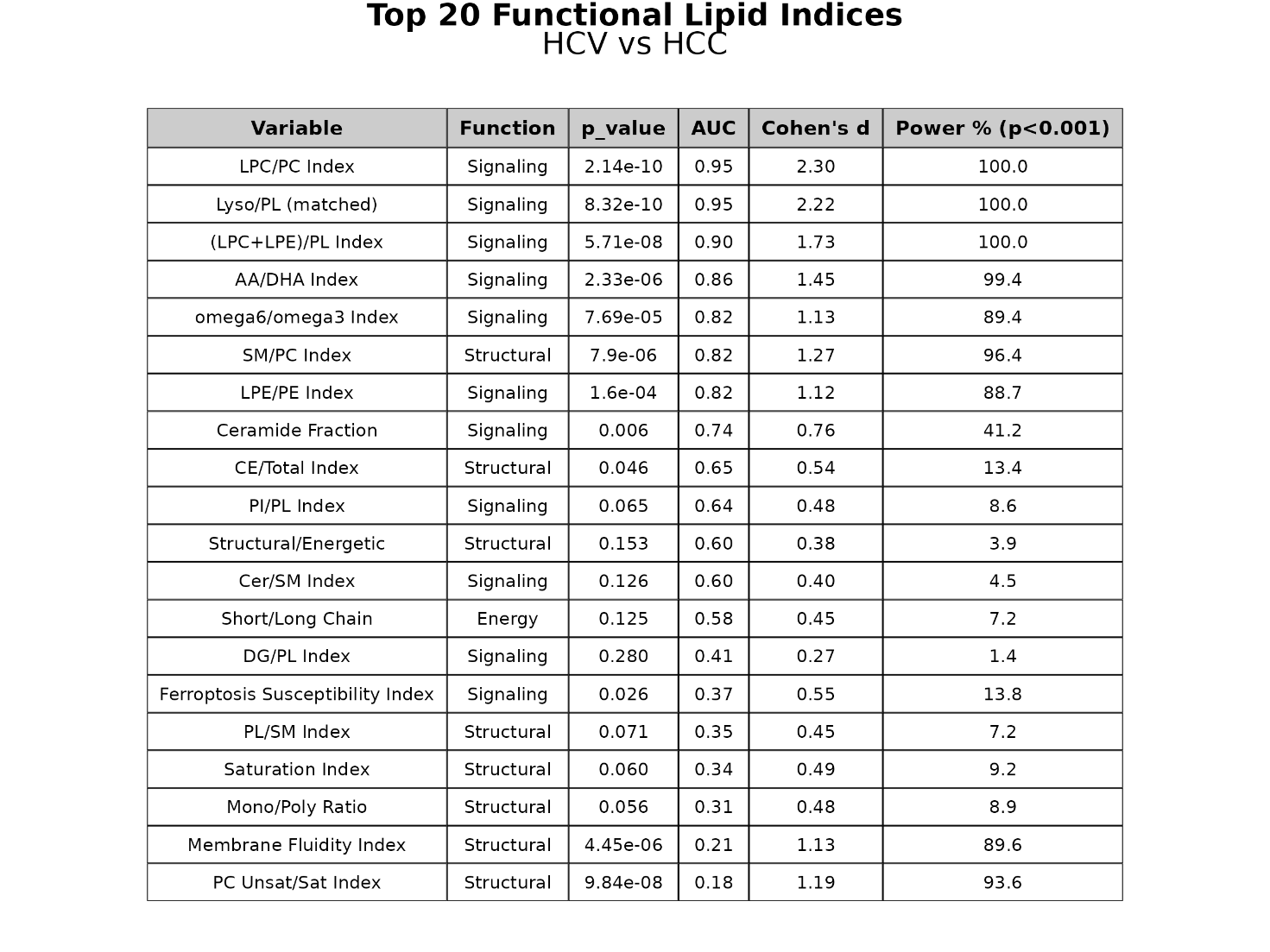


Figure S6: Top 20 functional lipid indices discriminating HCV from HCC. Table of the 20 highest-ranking FLA indices reporting Function, p-value, ROC AUC, Cohen’s d, and Power. Indices with AUC < 0.5 indicate inverse polarity (higher in HCC); flipping the decision rule yields the complementary AUC (1 − AUC) with unchanged discriminative strength.

Consistent with Caponigro et al., signaling indices dominate the ranking. The strongest performers are LPC/PC and Lyso/PL (matched) (AUC ≈ 0.95; d ≥ 2.2), followed by (LPC+LPE)/PL and LPE/PE (AUC ≈ 0.90 and ≈ 0.82, respectively). Together these increases are consistent with increased PLA2-driven membrane turnover and enhanced inner/mitochondrial-membrane dynamics in HCV. Inflammatory balance indices (AA/DHA, omega-6/omega-3) also rank highly, consistent with a more pro-inflammatory lipid milieu in HCV.

Structural readouts point to more ordered/less fluid membranes in HCV: PC Unsat/Sat and the Membrane Fluidity Index show strong effects with inverse polarity (AUC < 0.5), while SM/PC is higher in HCV and directionally concordant. The Ceramide Fraction shows moderate discrimination (p = 0.006; AUC ≈ 0.74) but does not exceed the preset effect-size threshold in the volcano plot. Energy-related indices cluster near the origin and do not pass the combined filters, indicating that the dominant HCV–HCC differences are signaling/structural rather than storage-centric.


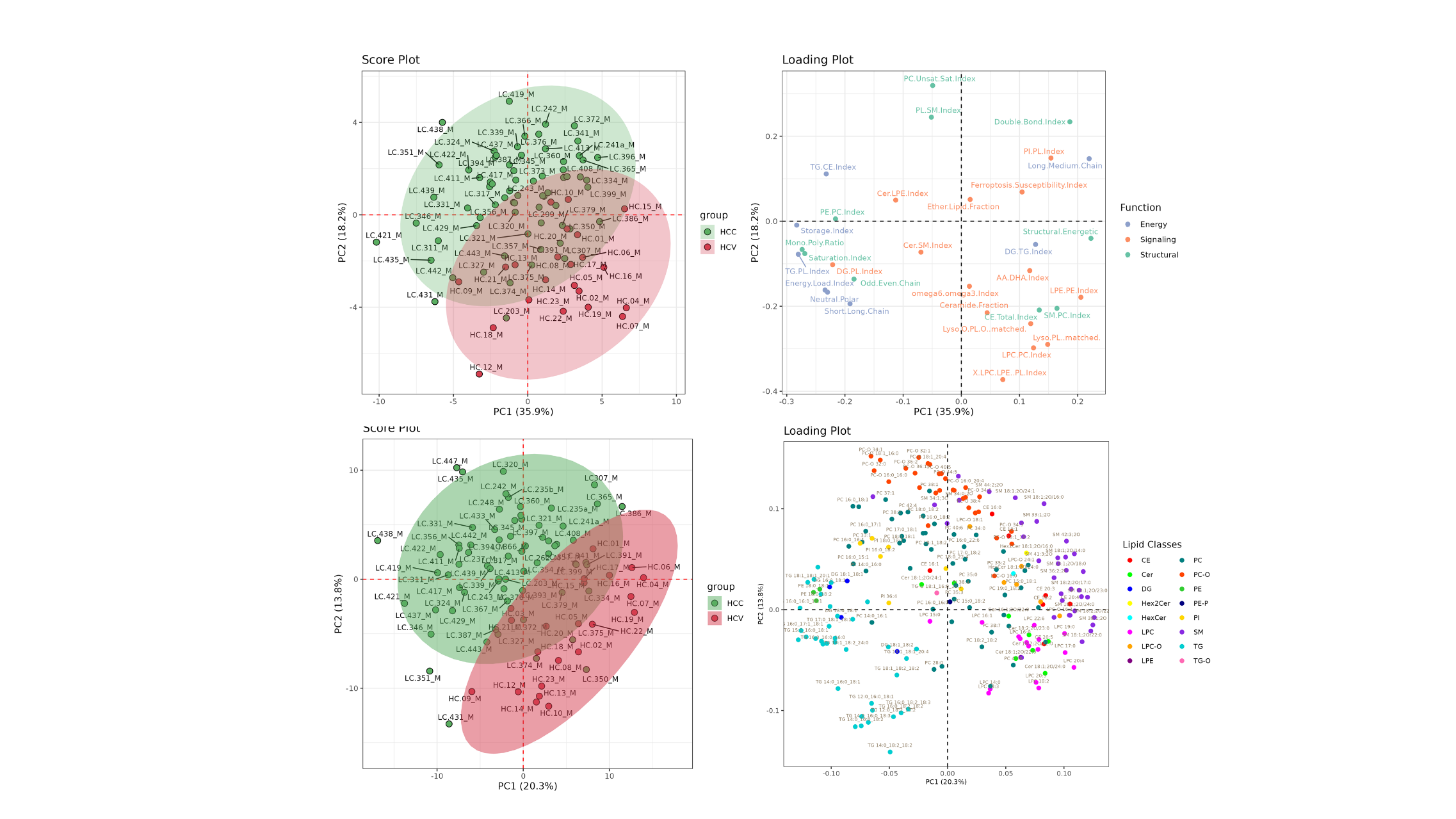


Figure S7: PCA on functional indices versus molecular species. Top: PCA computed on the matrix of functional indices (score plot left; loading plot right). Bottom: PCA computed on molecular lipid species from the same samples (score left; loading right). Both analyses use identical preprocessing (centred and auto scaled). Score plots show 95% confidence ellipses for groups; axes report the percentage of explained variance. In this dataset, PC1 explains 35.9% of the variance with indices and 20.3% with molecular species, illustrating how index-based features sharpen group separation and yield more interpretable loadings (indices coloured by function; species coloured by lipid class).

Moreover, when applying PCA to the molecular species to explain the variations (Figure S7), the first principal component accounts for 20.3% of the variance, compared to 35.9% when computed on indices. These results once again confirm that indices enhance the analytical power and provide more interpretable information.

Overall, functional indices dampen single-species noise while retaining statistical power and mechanistic interpretability. This re-analysis confirms the lysophospholipid depletion in HCC reported by Caponigro et al. and reframes it within a functional taxonomy linking membrane remodeling, inflammation, and organelle turnover, yielding robust, clinically relevant candidates for HCC detection in HCV-positive populations.

References

Caponigro,V. *et al.* (2023) Integrated plasma metabolomics and lipidomics profiling highlights distinctive signature of hepatocellular carcinoma in HCV patients. *J Transl Med*, **21**, 918.

Dean,J.M. and Lodhi,I.J. (2018) Structural and functional roles of ether lipids. *Protein Cell*, **9**, 196–206.

Jové,M. *et al.* (2023) Ether Lipid-Mediated Antioxidant Defense in Alzheimer’s Disease. *Antioxidants (Basel)*, **12**, 293.

Perez,M.A. *et al.* (2022) Ether lipid deficiency disrupts lipid homeostasis leading to ferroptosis sensitivity. *PLoS Genet*, **18**, e1010436.
